# HMO-primed bifidobacteria exhibit enhanced ability to adhere to intestinal epithelial cells

**DOI:** 10.3389/fmicb.2023.1232173

**Published:** 2023-12-15

**Authors:** Clodagh Walsh, Rebecca A. Owens, Francesca Bottacini, Jonathan A. Lane, Douwe van Sinderen, Rita M. Hickey

**Affiliations:** ^1^Teagasc Food Research Centre, Moorepark, Cork, Ireland; ^2^Health and Happiness Group, H&H Research, Cork, Ireland; ^3^APC Microbiome Ireland and School of Microbiology, University College Cork, Cork, Ireland; ^4^Department of Biology, Maynooth University, Maynooth, Ireland; ^5^Biological Sciences and ADAPT Research Centre, Munster Technological University, Cork, Ireland

**Keywords:** HMO, *Bifidobacterium*, probiotics, prebiotics, colonization, breastmilk, hIECs

## Abstract

The ability of gut commensals to adhere to the intestinal epithelium can play a key role in influencing the composition of the gut microbiota. Bifidobacteria are associated with a multitude of health benefits and are one of the most widely used probiotics for humans. Enhanced bifidobacterial adhesion may increase host-microbe, microbe-nutrient, and/or microbe-microbe interactions, thereby enabling consolidated health benefits to the host. The objective of this study was to determine the ability of human milk oligosaccharides (HMOs) to enhance bifidobacterial intestinal adhesion *in vitro*. This study assessed the colonisation-promoting effects of HMOs on four commercial infant-associated *Bifidobacterium* strains (two *B. longum* subsp. *infantis* strains, *B. breve* and *B. bifidum*). HT29-MTX cells were used as an *in vitro* intestinal model for bacterial adhesion. Short-term exposure of four commercial infant-associated *Bifidobacterium* strains to HMOs derived from breastmilk substantially increased the adherence (up to 47%) of these probiotic strains. Interestingly, when strains were incubated with HMOs as a four-strain combination, the number of viable bacteria adhering to intestinal cells increased by >90%. Proteomic analysis of this multi-strain bifidobacterial mixture revealed that the increased adherence resulting from exposure to HMOs was associated with notable increases in the abundance of sortase-dependent pili and glycosyl hydrolases matched to *Bifidobacterium bifidum*. This study suggests that HMOs may prime infant gut-associated *Bifidobacterium* for colonisation to intestinal epithelial cells by influencing the expression of various colonization factors.

## 1 Introduction

Bifidobacterial strains are the most abundant colonizers in the gut of breast-fed infants, accounting for 50%–90% of the total bacterial population detected in their faeces (Milani et al., 2017). The successful adaptation of bifidobacteria to the infant gut has been largely attributed to their large arsenal of carbohydrate-active enzymes, which allows specific bifidobacterial strains to utilise breast-milk derived glycans such as human milk oligosaccharides (HMOs). HMOs act as prebiotics [defined as “a substrate that is selectively utilized by host microorganisms conferring a health benefit” (Gibson et al., 2017)] by promoting growth of beneficial intestinal bacteria and thereby generating short-chain fatty acids which are critical for gut health (Pokusaeva et al., 2011). Due to their historical use and health-promoting properties, bifidobacterial strains are some of the most widely used and clinically documented probiotics. As probiotics, bifidobacteria contribute to host health through modulation of the intestinal microbiota, promotion of immune system development, maintenance of mucosal barrier integrity, and resistance to pathogenic colonization (Gareau et al., 2010). However, in order for bifidobacterial probiotics to impart their beneficial role, they not only need to compete with other gut commensals for nutrient acquisition, but also need to survive gastrointestinal transit and stably colonize the gastrointestinal tract (Morrin and Hickey, 2020). Adhesion to intestinal cells and/or mucus should therefore act as a key selection criterion when developing probiotic-containing products.

Recent studies have identified a plethora of bacterial features that have been shown to be involved in host–microbe interactions (Chang and Kao, 2019; Viljoen et al., 2020; Xiao et al., 2021). In this regard, cell surface components, termed adhesins, play an important role in allowing bifidobacteria to adhere to and interact with intestinal epithelial cells (Westermann et al., 2016). Many microbial adhesins display lectin-like properties and bind corresponding glycan receptors on the surface of the host cells via carbohydrate-recognition domains (CRDs). These interactions can be an important determinant of host and tissue tropism. Bacterial adhesion to human intestinal cells often involves the assembly of elongated, submicroscopic structures called pili (resembling “threads”) or fimbriae (also known as short attachment pili or “hairs”) (Nizet et al., 2017). Mounting evidence suggests that numerous bifidobacterial strains encode and express pilus-like structures (Foroni et al., 2011; O'Connell Motherway et al., 2011, 2019; Westermann et al., 2016). In recent years, several studies have indicated that certain milk components could enhance the adhesion ability of bifidobacterial strains by influencing the expression of these bacterial adhesins. For instance, González and colleagues found that the FimA fimbrial subunit and sortase-like fimbria-associated protein (involved in the assemblage of fimbriae) were highly upregulated during growth of *Bifidobacterium longum* in human milk and formula milk (González et al., 2008). Researchers have hypothesized that one such milk component contributing to this enhanced adhesion is the prebiotic fraction in human milk; HMO. An *in vitro* study by Chichlowski et al. demonstrated that exposure of *B. infantis* ATCC 15697 to breastmilk-derived HMO significantly increased adherence of the strain to HT-29 intestinal cells (Chichlowski et al., 2012). More recently, findings from Zhang et al. indicate that 2′-fucosyllactose (2′-FL), typically the most abundant HMO in breastmilk, promotes *B. bifidum* DNG6 adhesion to Caco-2 cells through increased expression of genes encoding adhesion proteins (Zhang et al., 2020). Likewise, incubation of *B. infantis* ATCC 15697 in the presence of 3′-siallylactose (3′-SL) and 6′-siallylactose (6′-SL), two predominant acidic oligosaccharides found in the milk of most mammals, substantially increased the adherence of the bacteria to HT-29 cells (Kavanaugh et al., 2013). Subsequent transcriptomic analysis revealed that this increased adherence was associated with heightened levels of tight-adherence protein TadE expression. This protein plays a pivotal role in orchestrating the assembly of type IVb pili surface appendages (Kavanaugh et al., 2013).

The objective of the current study was to assess the ability of HMOs to enhance adhesion of a community of bifidobacterial strains to mucus-secreting intestinal cells. As outlined by Xiao et al. the majority of current studies assessing colonization capabilities of probiotic strains have been performed using allochthonous strains (i.e., those isolated from fermented foods or plants and then studied in humans) and therefore do not perform well in their new ecosystem (Xiao et al., 2021). It is suggested that, if the goal is stable colonization, authochthonous strains (i.e., those of human origin) should instead be examined (Xiao et al., 2021). The present study focused on four autochthonous bifidobacterial strains of human origin that are of commercial interest due to their historical employment as ingredients in functional foods and their clinically documented health benefits (Giannetti et al., 2017; Wong et al., 2019; Xiao et al., 2019). Here, we investigated the adhesive properties of *Bifidobacterium bifidum* R0071, *Bifidobacterium infantis* R0033, *Bifidobacterium breve* M-16 V, and *Bifidobacterium longum* subsp*. infantis* (*B. infantis*) M-63, which are all shown to elicit potent immunomodulatory and protective effects *in vivo* (Giannetti et al., 2017; Wong et al., 2019; Xiao et al., 2019). Genomic mining and proteomic analyses were subsequently performed to understand the mechanisms underlying bifidobacterial adherence to intestinal cells. Previous work by our group has shown that positive co-operation between these bifidobacterial strains in utilising HMOs as a carbohydrate source resulted in higher cell numbers (Walsh et al., 2022). Given the evolution of these strains in a HMO-rich environment, we hypothesised that HMOs would not only influence the selective growth of these strains in a community setting, but also their specific adhesive ability. This study provides further insights into the role that HMOs play in selectively promoting bifidobacterial colonization in the infant gut and corroborates the notion that oligosaccharides acting as prebiotics also have the potential to enhance bifidobacterial adhesion.

## 2 Materials and methods

### 2.1 Generation of HMO-enriched fraction

Pooled human milk samples (of unknown secretor status) were kindly donated by Irvinestown Human Milk Bank (Co. Fermanagh, Ireland) and stored at −80°C on arrival. Briefly, lipids and proteins were removed from breastmilk as previously described (Quinn et al., 2018) before separation of lactose and HMO components using size-exclusion chromatography (BioGel P2, 92 × 5 cm; Bio-Rad Laboratories, Inc., Hercules, CA, United States). Peptide-free (quantified using Pierce colorimetric peptide assay) and low-trace lactose [quantified using high pH anion exchange chromatography (HPAEC) with pulsed amperometric detection (Dionex Corporation, Sunnyvale, CA, United States)] fractions were pooled and freeze-dried. The resultant HMO-enriched fraction was stored at 4°C prior to use in experiments. HPAEC analysis indicated that low levels of 2′-fucosyllactose (2′-FL) were found in the HMO-enriched fraction, likely due to the purification process where 2′-FL and lactose fractionated together. Therefore, the 2′-FL which had been eliminated during the size exclusion process was resubstituted using commercial 2′-FL (Friesland Campina), as previously described (Walsh et al., 2022), to give a powder representative of secretor HMO (designated S-HMO). S-HMO was used in adhesion studies at concentrations outlined in previous studies from our group (Walsh et al., 2022): final concentration of 5 g/L (3.8 g/L isolated HMO + 1.2 g/L 2′-FL). The composition of the S-HMO blend is shown in Supplementary Figure S1.

### 2.2 Epithelial cell line conditions

The human colonic adenocarcinoma cell line HT29-MTX [European Collection of Authenticated Cell Cultures (ECACC)] was used as a model of human intestinal epithelia. HT29-MTX cells were used for experiments at three different passages (passage numbers 38 to 41) to provide three biological replicates. Cells were routinely cultured in Dulbecco’s Modified Eagle’s Medium (DMEM) [10% Fetal Bovine Serum (FBS), 1% non-essential amino acids] (Merck, Darmstadt, Germany) in 75 cm^2^ tissue culture flasks at 37°C with humidified atmosphere (5% CO_2_). The cultures were passaged by detaching with trypsin when cells had reached approximately 90% confluence. Cells were seeded at a density of 1×10^5^ cells/mL into 12-well plates (Cellbind; Corning, New York, United States). HT29-MTX cells were used once 100% confluency was reached (approximately 5 × 10^6^ cells/well at day 12–14). Growth medium was changed every other day and supplemented with 2% FBS 24 h prior to use.

### 2.3 Bacterial strains and culture conditions

Commercial *Bifidobacterium* strains used in this study were obtained from Lallemand Health Solutions, France (*Bifidobacterium bifidum* R0071 and *Bifidobacterium infantis* R0033), and Morinaga Milk, Japan (*Bifidobacterium breve* M-16 V and *Bifidobacterium infantis* M-63). Strains were stored at −80°C in de Man-Rogosa-Sharpe (MRS) broth (Difco, BD, Ireland), supplemented with 50% (v/v) glycerol as a cryoprotectant. For routine experiments, strains were grown at 37°C under anaerobic conditions using MRS medium supplemented with 0.05% (wt/v) L-cysteine hydrochloride (Merck) and 0.01% (wt/v) mupirocin (Merck). Bacterial stocks were regularly checked for contamination by closely monitoring colony morphology during culturing. In addition, the bacterial stocks were routinely validated as being specific to their genera using bifidobacterial-specific PCR targeting the 16S region using primers g-Bifid-F (5′-CTCCTGGAAACGGGTGG-3′) and g-Bifid-R (5′-CTCCTGGAAACGGGTGG-3′) (Matsuki et al., 2004). Resultant amplicons were sequenced using Sanger sequencing.

### 2.4 Colonisation assays

#### 2.4.1 Priming of bifidobacterial strains

For experiments, bifidobacterial strains derived from −80°C stock cultures were streaked on MRS agar supplemented with 0.05% (wt/v) L-cysteine HCl and incubated for 48 h at 37°C under anaerobic conditions. Single colonies of each strain were subsequently inoculated in supplemented MRS broth and sub-cultured twice before use in subsequent experiments. To obtain working cultures, supernatant of overnight culture was removed by centrifugation, after which the bacterial cell pellet was washed with phosphate buffer saline (PBS) (Merck) and resuspended in tissue culture media (2% FBS) such that the OD_600_ of each bacterial culture was ~0.4 (corresponding to ~5×10^7^ CFU/mL, as determined by plate counts). The following bacterial combinations were assessed: single cultures of *B. bifidum* (R0071), *B. infantis* (R0033), *B. breve* (M-16 V), *B. infantis* (M-63) and a mixture of all four bifidobacterial strains (4Bif) containing equal volumes of each~5×10^7^ CFU/mL bacterial culture prepared as described above Bacterial suspension was added at a 1:1 ratio to DMEM tissue culture media (2% FBS) supplemented with 10 mg/mL of S-HMO or 2′-FL. Therefore, the final concentration of bacteria and carbohydrate at initiation of incubation was 2.5×10^7^ CFU/mL and 5 mg/mL, respectively. Bacteria suspended in non-supplemented DMEM tissue culture media (without S-HMO or 2′-FL) was used as a control. Bacteria were exposed to oligosaccharides at 37°C under anaerobic conditions for 1 h before removal of residual oligosaccharides by centrifugation. The bacterial pellet was washed three times using PBS and centrifugation before resuspending in non-supplemented DMEM media prior to use in the assays.

#### 2.4.2 Bacterial exposure to eukaryotic cells

Eukaryotic cells were washed twice with PBS, and 0.5 mL of bacterium:medium suspensions were added to the appropriate wells (corresponding to approximately 10–20 bacterial cells per eukaryotic cell). Bacteria were exposed to eukaryotic cells for 2 h at 37°C under anaerobic conditions, after which wells were washed three times with PBS to remove non-adherent bacteria, and then lysed with 0.1% Triton X100 (Merck) for 30 min. Bacteria within extracted lysates were quantified using two methods.

Firstly, viable bacteria adhering to cells were enumerated by serially diluting lysates and spread-plating (in triplicate) on MRS plates. Aliquots of the experimental inocula were retained, diluted, and plated to determine original CFU/mL. Results were expressed as adherent bacteria as a percentage of the original inoculum, thereby accounting for variations in the original inocula between treatment groups. Percentage adherent = [CFU/mL of recovered adherent bacteria/ CFU/mL of inoculum] × 100. Comparisons between strains and treatments groups were calculated as fold change. Fold change in adherence = Percentage adherent treatment group/ percentage adherent control group.

Secondly, bacterial numbers were determined using quantitative PCR (qPCR) as previously described (Walsh et al., 2022). Bacterial DNA was extracted using GenElute Bacterial Genomic DNA Kit (Merck) and quantified using a 480 Lightcycler platform (Roche Applied Science, Penzberg, Germany) using the following program: 95°C for 5 min followed by 40 cycles of 95°C for 10 s, 58°C for 20 s and 72°C for 30s. Species within the co-culture were enumerated using primers targeting the 16S rDNA gene region. For quantification of all *Bifidobacterium* strains present in samples, the primer pairs Bifid-F and g-Bifid-R were used. To determine *B. bifidum* cell numbers, primer pairs BiBIF-1 (5′-CCACATGATCGCATGTGATTG-3′) and BiBIF-2 (5′-CCGGATGCTCCATCACAC-3′) were employed. *B. breve* cells were quantified using primer pair BiBRE-1 (5′-CCACATGATCGCATGTGATTG-3′) and BiBRE-2 (5′-ACAAAGTGCCTTGCTCCCT-3′), while *B. infantis* cell numbers were determined using primer pair BiINF-1 (5′-TTCCAGTTGATCGCATGGTC-3′) and BiINF-2 (5′-GGAAACCCCATCTCTGGGA-3′). The copy-number of a given species in co-culture was evaluated by comparing the cycle threshold (Ct) values with those from a standard curve of known copy number. Standard curves (10^9^ to 10^2^ copy number/ μL) were established by performing 1 in 10 serial dilutions of 16S rDNA of the specified bifidobacterial species (in the case of *B. infantis* strains, M-63 was used as the reference rDNA). The cell numbers of species within co-culture (denoted by bifidobacteria/mL) were then deduced by comparing these copy number values with copy numbers of standards with known cell numbers (as determined by viable count assessment). Cell numbers were calculated according to the formula (Quigley et al., 2013):


$$\frac{\left(C/\mathrm{\mu l}\right)\left(TV\right)}{TCN}\times \mathrm{Tcfu}/\mathrm{mL}=\mathrm{bifidobacteria}/\mathrm{mL}\;\left(S\right)$$

Where; C/μL = Copy number/μL, TV = template volume (μL), TCN = total copy number of the standard used, T cfu/mL = total cell number of standard used, and bifidobacteria/mL(S) = cell number of test sample. Samples were run in triplicate, while standards and negative controls (where template DNA was replaced with PCR grade water) were run in duplicate.

### 2.5 Whole genome sequencing assembly and annotation

Following growth of R0071, R0033, M-16 V and M-63 as described in Section 2.3 above, bacterial cells were harvested, lysed, and total bacterial DNA was isolated. Genome sequences of the bifidobacterial strains were generated by Macrogen Inc. (Seoul, South Korea) using Pacific Biosciences SMRT RSII technology. The raw sequencing reads were *de novo* assembled using the Hierarchical Genome Assembly Process (HGAP) protocol RS_Assembly.2 implemented in the SMRT Analysis software v.2.3 with default parameters^1^ as previously described (Bottacini et al., 2017).

Open Reading Frame (ORF) prediction and automatic annotation was performed using Prodigal v2.0^2^ for gene predictions. BLASTP v2.2.26 (cut-off E-value of 0.0001) was used for sequence alignments against a combined bifidobacterial genome-based database, and MySQL relational database to assign annotations. Predicted functional assignments were manually revised and edited using similarity searches against the non-redundant protein database curated by the National Centre for Biotechnology Information^3^ and PFAM database,^4^ which allowed for *in silico* characterization of hypothetical proteins. GenBank editing and manual inspection was performed using Artemis v18.^5^ Transfer RNA genes were identified employing tRNAscan-SE v1.4 and ribosomal RNA genes were detected based on the software package Rnammer v1.2 (38) supported by BLASTN v2.2.26.

### 2.6 Prediction of colonisation capabilities

Scientific literature databases including PubMed, Web of Science, UCC library, and Google Scholar were searched for studies providing mechanistic insight into probiotic colonization and survival in the gastrointestinal tract. A list of probiotic (*Lactobacillus* and *Bifidobacterium* genera) colonisation-related features (Supplementary Table S3) was then compiled from these studies which included gene-trait matching, transcriptomic, proteomic, and knock-out studies (Tanaka et al., 2000; Walter et al., 2005, 2007, 2008; Bron et al., 2007; Guglielmetti et al., 2008; Shkoporov et al., 2008; Gueimonde et al., 2009; Kankainen et al., 2009; Foroni et al., 2011; Frese et al., 2011, 2013; O'Connell Motherway et al., 2011; Sims et al., 2011; Fanning et al., 2012; González-Rodríguez et al., 2012; Turroni et al., 2013; Christiaen et al., 2014; Goh and Klaenhammer, 2014; Liu et al., 2014; Wilson et al., 2014; Call et al., 2015; Ganesh et al., 2018; Ishikawa et al., 2021). A Blast search was then performed (e-value cutoff of 1e-5, query cover cutoff of 50%) of the database against the bifidobacterial genomes of interest. Percent identity was recorded for each bifidobacterial strain of interest and classified as “low identity” = 30%–50%, “moderate identity” = 50%–70%, and “high identity” = 70%–100%.

The bifidobacterial sequences were also searched for sortase-dependent proteins since these proteins can play a pivotal role in adhesion and mucin-degradation. The bifidobacterial strains were surveyed for features containing the following sequence:

1. a signal peptide at the N-terminus
2. a pentaglycine recognition motif
  - LPXTG in the case of sortase A- and sortase C-dependent proteins
  - [L/I/V][S/A]XTG in the case of sortase E-dependent proteins
3. followed by a hydrophobic membrane-spanning region (verified using Protscale https://web.expasy.org/protscale/ and DeepTMHMM v1.0.13 https://dtu.biolib.com/DeepTMHMM)
4. positively charged residues at the C-terminus.

### 2.7 Proteomic analysis of colonisation-related factors

#### 2.7.1 Sample preparation

Label-free quantitative proteomic analysis was carried out on the four-strain bifidobacterial mixture with/without S-HMO pre-treatment and after exposure to the HT29-MTX cell line. Sample groups included S-HMO-treated (S-HMO^+^cells^+^), and untreated bifidobacteria (S-HMO^−^cells^+^), following incubation with cells, and untreated bifidobacterial control (S-HMO^−^ Cells^−^) without incubation with HT29-MTX cells. Biological replicates of treated and control cells were prepared (*n* = 3 per condition).

HT29-MTX cells were seeded in 6-well plates and cultured until 100% confluence was reached. A bifidobacterial mixture containing equal amounts of *B. bifidum* R0071, *B. infantis* R0033, *B. breve* M-16 V, & *B. infantis* M-63 was prepared as outlined in section 2.4. This bacterial mixture was then incubated for 2 h in non-supplemented serum-free phenol-red free DMEM with and without HMO. For experiments, HT29-MTX cells were washed with PBS and treated with 3 mL bacterial solutions. The plates were then incubated at 37°C for 2 h in a humidified atmosphere (5% CO_2_). As a control, bacteria were incubated under the same conditions in DMEM without HT29-MTX cells. After 2 h incubation, medium containing the treated bacteria was aspirated (Wei et al., 2014). Thereafter, bacteria were pelleted, washed three times in PBS and immediately frozen in liquid nitrogen. Adherent bacteria were also extracted from the system by detaching HT29-MTX cells with ice-cold PBS. Harvested cells were immediately frozen in liquid nitrogen. All samples were stored at −70°C until protein extraction was performed.

#### 2.7.2 Protein extraction and digestion

Cold lysis buffer (0.1 M TrisHCl, 10% glycerol, 20 mM EDTA, 50 mM NaCl, 1 mM PMSF, 1 μg/mL, pH 7.5) was added to each cell pellet and sonicated using a MS72 probe (3 × 10 s, cycle 6, 20% power), and placed on ice between each sonication round. An aliquot of each lysate was clarified by centrifugation and a Bradford protein assay (Bio-Rad) was used to determine the protein concentration. Total lysates (without clarification) were adjusted to 0.25 mg/mL protein concentration with 50 mM ammonium bicarbonate and heated at 95°C, for 5 min. Samples were reduced using dithiothreitol (DTT; 5 mM final, 15 min), alkylated using iodoacetamide (IAA; 15 mM final, 20 min, dark) and subjected to overnight trypsin digestion at 37°C (trypsin: 8 μg/mL final) in the presence of ProteaseMax (0.01% (w/v)). Digestion was stopped by addition of trifluoroacetic acid (1% (v/v)) and samples were dried under vacuum. Digests were resuspended in 0.5% (v/v) TFA prior to clean-up using C18 ZipTips (Millipore) according to manufacturer’s instructions. Samples were dried under vacuum and stored at −70°C until LC–MS/MS analysis.

#### 2.7.3 Label-free quantitative (LFQ) proteomic analysis

Peptide extracts (0.75 μg) were analysed by LC–MS/MS using the Thermo Q-Exactive mass spectrometer coupled to a Dionex Ultimate 3,000 RSLCnano, using a 2.25 h method for separation on an Easy-Spray PepMap™ column (50 cm × 75 μm, 2 μm particles). Samples were searched against a composite database containing the proteomes of R0071, M-16 V, and M-63, appended with the *Homo sapiens* proteome (downloaded from Uniprot 15/02/2019) using MaxQuant (v 1.6.2.10) with MaxLFQ algorithm included for protein ratio characterisation (Cox et al., 2014) and 1% FDR applied. Perseus (v 1.6.2.2) was used for data analysis (Tyanova et al., 2016). Proteins were filtered to exclude single peptide identifications and proteins matching a reverse or a contaminants database. Proteins were retained for analysis only if detected in at least two biological replicates from either comparator group. Qualitative results were based on unique protein detection in at least two biological replicates in a condition, with absence of detection in all replicates of the comparator set. Quantitative and qualitative results were combined prior to functional characterization.

#### 2.7.4 Statistical analysis

Statistical analyses for adhesion assays were performed using technical triplicate data (three wells of each biological replicate) from biological triplicate experiments (across three successive passages of HT29-MTX cells). Graphs were created using PRISM v8 (GraphPad, La Jolla, CA) and were plotted as mean ± sd. Two-way univariate analysis of variance (ANOVA) and post-hoc Tukey tests were performed on bifidobacterial adhesion levels with the strain and carbohydrate source as factors. Statistical significance was accepted as *p* ≤ 0.05. For proteomic data Perseus was used for statistical analysis, with quantitative results composed of proteins with a significant change in abundance [*p* < 0.05 (Student’s t-test), fold change ≥2]. The distribution and intersection of proteins detected across strains and carbohydrate source was assessed using Venn Diagrams. The Venn Diagram was initially constructed using the Draw Venn Diagram tool at Bioinformatics & Evolutionary Genomics^6^ and reformatted thereafter using Microsoft Powerpoint.

## 3 Results

### 3.1 Single strain and multi-strain adherence to HT29-MTX

Adherence of probiotic bacteria was evaluated using HT29-MTX human tumorigenic cell lines to mimic bifidobacterial adhesion to intestinal epithelial cells (IECs). Bifidobacterial strains were incubated with various carbohydrates (glucose, 2′-FL, or S-HMO) after which their ability to adhere to HT29-MTX cells was determined. The “secretor-HMO” (S-HMO) used in the experiments was obtained through the purification of pooled breast milk using gel-filtration chromatography. Initial screenings revealed that during the size exclusion process using Bio-Gel polyacrylamide beads, lactose and 2′-FL were found to co-fractionate. Consequently, all fractions containing high levels of lactose and 2′-FL were excluded from the final HMO-enriched mixture. While the most abundant α1,2-fucosylated HMO was removed from the resulting powder, the preparation still included other HMOs containing H-antigens such as difucosyllactose (DFL), lacto-N-fucopentaose I (LNFP I), and lacto-N-difucohexaose I (LNDFH I) (see Supplementary Figure S1). To create an ingredient representative secretor HMO, the previously eliminated 2′-FL was then reintroduced (usually a commercially produced 2′-FL) and designated as S-HMO.

**Figure 1:**
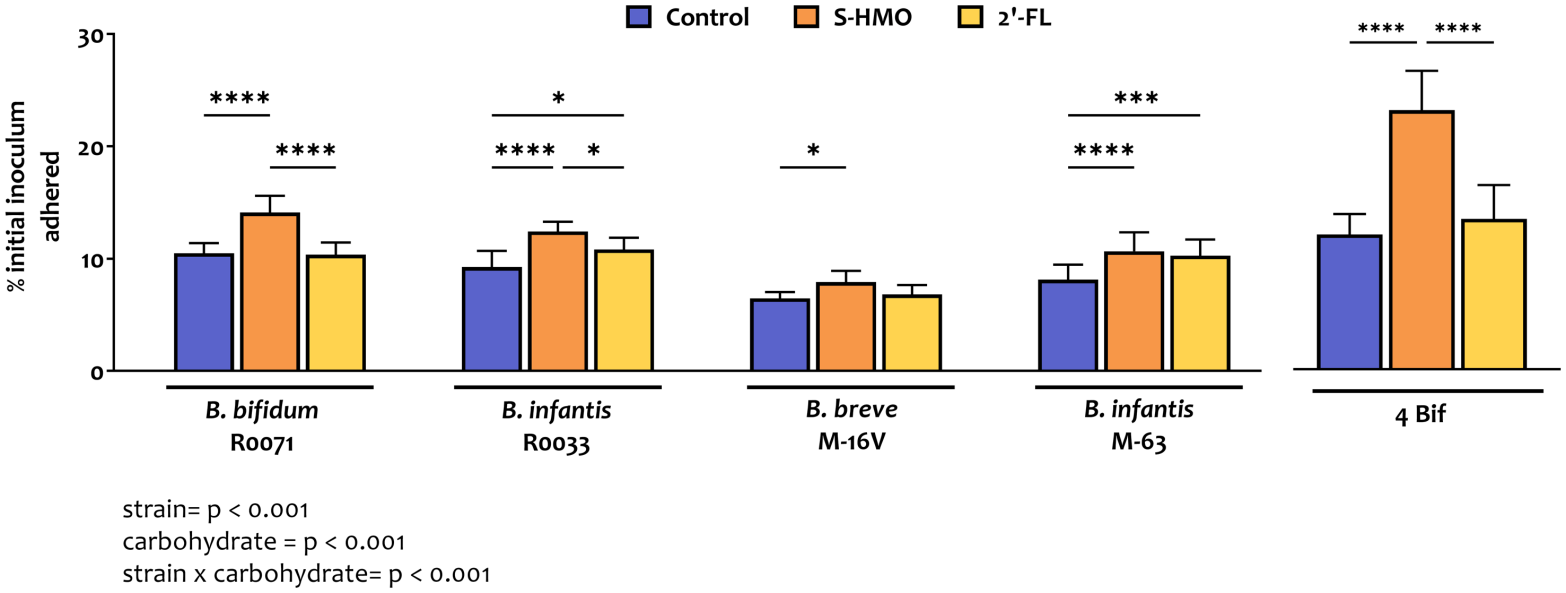
Levels of bifidobacterial adherence to HT29-MTX cells following pre-exposure to glucose control, breastmilk-derived human milk oligosaccharides (S-HMO), and 2′-fucosyllactose (2′-FL). Adherent bacteria are plotted as a percentage of the original inoculum. Percentage adherent = [CFU/mL of recovered adherent bacteria/ CFU/mL of inoculum] × 100. R0071 = *Bifidobacterium bifidum* R0071, R0033 = *Bifidobacterium infantis* R0033, M-16 V = *Bifidobacterium breve* M-16 V, M-63 = *Bifidobacterium infantis* M-63, 4Bif = four *Bifidobacterium* mixture. Analysis was calculated using technical triplicate data from biological triplicate experiments and data are means ± SD. Univariate analysis of variance (ANOVA) and post-hoc Tukey tests were performed to determine the significant differences between the groups (*p < 0.05, **p < 0.01, ***p < 0.001, ****p < 0.0001). Results of ANOVA are as outlined in figure where row factor = bifidobacterial strain(s), column factor = carbohydrate source, and interaction = bifidobacterial strain × carbohydrate source.

As shown in Figure 1 and Supplementary Table S1, adhesion of bifidobacterial strains varied significantly depending on strain and carbon source. The lowest levels of adherence were observed with *B. breve* M-16 V in control group where glucose was the sole carbon source (7.2 ± 0.6% of original inoculum), while highest levels of adherence were observed with strain *B. bifidum* R0071 after exposure to S-HMO (13.8 ± 1.4% of original inoculum). Across all treatment groups, *B. bifidum* R0071 and *B. infantis* R0033 demonstrated significantly higher adherence when compared to *B. breve* M-16 V (Supplementary Table S1).

Addition of 2′-FL resulted in a marked increase in the adhesive ability of the *B. infantis* strains, as represented by a 1.26 fold increase in adhesion of M-63 (*p* = 0.0007) and a 1.15 fold increase in adhesion of R0033 (*p* = 0.0456) when compared to treatment of these strains in control media (Figure 1). Exposure of *B. bifidum* R0071 and *B. breve* M-16 V to 2′-FL had no significant impact on the adhesion of these strains to the cell line. However, exposure of each of the four *Bifidobacterium* strains to S-HMO significantly increased adherence to HT29-MTX cells by an average of 1.31 fold (*vs* control). The HMO-induced promotion of adhesion was observed, in particular, for *B. bifidum* R0071 which demonstrated significantly higher adhesion following S-HMO treatment when compared to the other strains (vs. R0033 *p* = 0.0170, vs. M-16 V and M-63 *p* < 0.0001, Supplementary Table S1). The adhesive ability of R0071 increased 1.37 fold after S-HMO treatment when compared to adhesion of the strain in control conditions (*p* < 0.0001). Interestingly, we also observed higher adhesion levels of *B. infantis* R0033 when compared to its species counterpart *B. infantis* M-63 following exposure of the strains to S-HMO (*p* = 0.0118).

**Figure 2:**
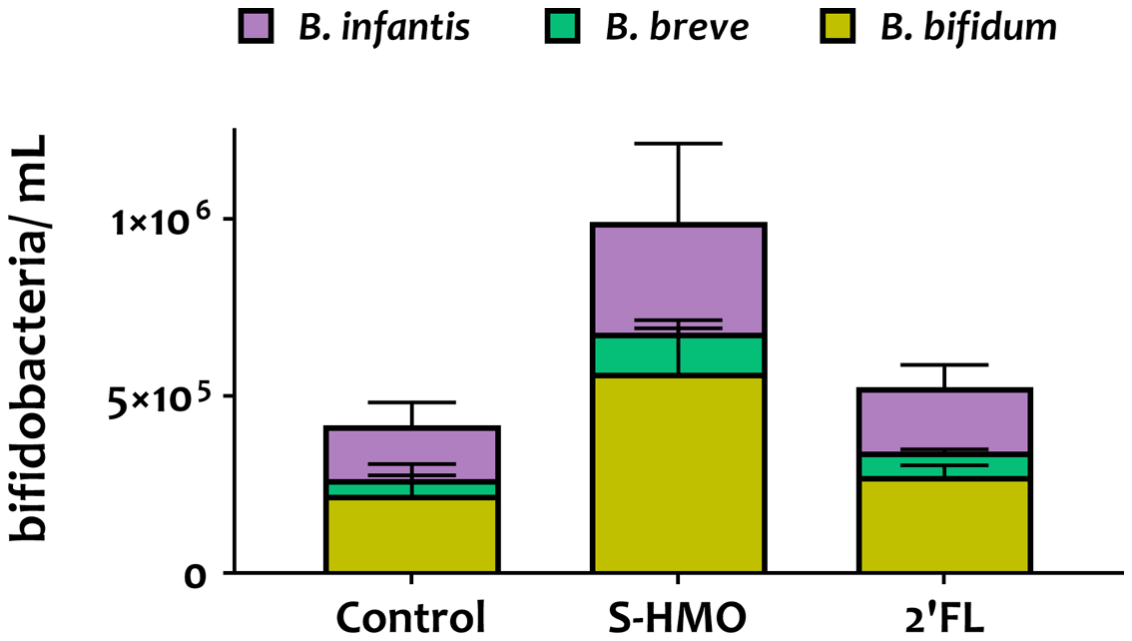
qPCR analysis of the adhered *Bifidobacterium* mixture (equal parts *Bifidobacterium bifidum* R0071, *Bifidobacterium infantis* R0033, *Bifidobacterium breve* M-16 V, *Bifidobacterium infantis* M-63) following pre-exposure to glucose control, breastmilk-derived human milk oligosaccharides (S-HMO), and 2′-fucosyllactose (2′-FL). Species within the bifidobacterial mixture were quantified using primers targeting the 16 s gene region of each species. Analysis was calculated using technical triplicate data from biological triplicate experiments and data are means ± SD.

To assess whether a probiotic combination represents better colonists than single strains, the adhesive capacity of a bifidobacterial mixture containing equal amounts of all four strains was assessed using HT29-MTX cells. Substantial levels of bacterial adhesion occurred with this bifidobacterial consortium (Figures 1, 2). Notable increases in adhesion were observed in the control group when compared to colonization by single strains, although these increases were not significant in the case of *B. bifidum* R0071 (11.9 ± 1.8% in co-culture versus 10.5 ± 0.9% in *B. bifidum* R0071 alone, Supplementary Table S1). However, significant increases in adherence levels of the four-strain mixture were observed following S-HMO-exposure when compared to treatment with control media (Figure 1). A large proportion of the original inoculum (23.0 ± 3.5%) was found to have adhered to intestinal cells following treatment with HMOs, which corresponds to a 1.92 fold increase when compared to the non-supplemented control (*p* < 0.0001, Supplementary Table S1).

Quantitative PCR analysis targeting the 16S rRNA-encoding region allowed for quantification of cell numbers of each species within the four strain combination but did not distinguish between the two *B. infantis* strains. Nonetheless, qPCR analysis provided insights into the microbial composition of the adhered bacteria (Figure 2). Across all treatments, *B. bifidum* occupied the largest proportion of adhered bacteria accounting for 53.0, 57.8, and 52.3% in control, S-HMO, and 2′-FL treatment groups, respectively (Figure 2). As shown in Supplementary Table S2, significantly higher levels of adhered *B. bifidum* were detected following S-HMO exposure, when compared to the two other species present within the bifidobacterial combination (vs. *B. breve p* < 0.0001, vs. R0033 *p* = 0.0157).

### 3.2 Mining of bifidobacterial sequences for colonization-related features

**Figure 3:**
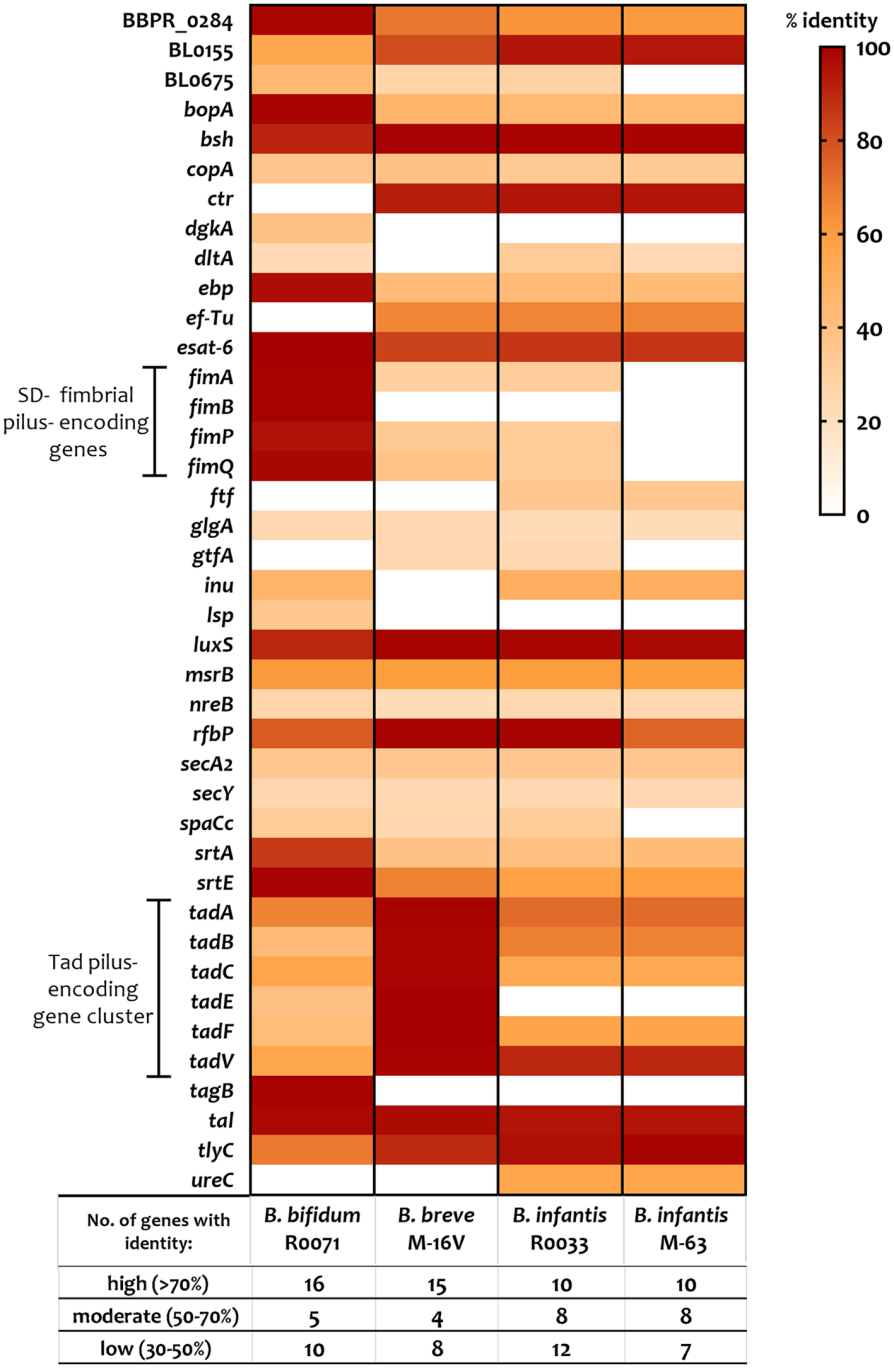
Percentage identity of genes predicted to be involved in probiotic survival and persistence in the gut. Genes, including those associated with assemblage of sortase-dependent (SD-) fimbriae or Tad pili (as highlighted), were identified using a variety of sources including gene-trait matching, transcriptomic, proteomic, and knock-out studies (see Supplementary Table S3 for further information). Percent identity (classified as “low identity” = 30%–50%, “moderate identity” = 50%–70%, and “high identity” = 70%–100%) is based on BLAST searches using e-value cutoff of 1e-5 and query cover cutoff of 50%.

To elucidate the key factors that are responsible for the observed differences in bifidobacterial adherence to intestinal cells, the bifidobacterial genomes were surveyed for colonisation-related factors. A literature search for studies assessing probiotic colonisation was performed and resulted in identification of 45 genes with a potential role in *Lactobacillus* and *Bifidobacterium* survival and persistence in the gut (Supplementary Table S3). Thereafter, the genomes of *B. bifidum* R0071, *B. breve* M-16 V, *B. infantis* R0033, and *B. infantis* M-63 were searched for homologs of these genes. All bifidobacterial strains studied here harboured genes associated with microbe-host interactions (>50% coverage, e-value <1 e-5). Genes with high similarity (>70% identity) to bile salt hydrolase (*bsh*, shown to improve adhesion ability of *Lactobacillus plantarum*), transaldolase (*tal*, demonstrated to bind mucin in strains of *B. bifidum*), S-ribosylhomocysteine lyase (*luxS*, shown to be involved in biofilm production in *B. breve* UCC2003), early secretory antigen target 6-like protein (*esat*-6 [*BBPR*_0651], found to be highly induced in *B. bifidum* PRL2010 following HT29 exposure), and hemolysin-like protein (*tlyC1*, shown to be involved in bile tolerance in *B. longum* BBMN68) were identified across all four genomes as well as genes involved in exopolysaccharide production such as undecaprenyl-phosphate galactosephosphotransferase *rfbP* (Figure 3; Supplementary Table S3).

The *tad* locus [as first described for the bifidobacterial genera in UCC2003 (O'Connell Motherway et al., 2011)], which encodes for site-determining protein TadZ, ATPase TadA, integral membrane proteins TadB and TadC, pre-pilin precursor Flp, and the pseudopilins TadE and TadF was found to be moderately conserved across the infant bifidobacterial strains studied here (as highlighted in Figure 3). Notably, we identified clear homologs (>70% identity) of all genes within the UCC2003 *tad* locus in *B. breve* M-16 V at *BBREV*_0119 to *BBREV*_0125 (Supplementary Table S3; Figure 3). Genes with homology (>50% coverage, e-value <1 e-5) to the UCC2003 *tad* locus were also identified in *B. bifidum* R0071 (at gene region *BBIF_*1744-*BBIF_*1750), although the level of genomic similarity was low (30%–50% identity) for *tadB*, *tadE*, and *tadF* (Supplementary Table S3; Figure 3). The *B. infantis* strains (R0033 and M-63) were found to harbour genes with moderate genomic similarity (50%–70% identity) to *tadA*, *tadB*, *tadC*, and *tadF* (Supplementary Table S3; Figure 3). However, no homologs to *tadE* were identified in either *B. infantis* strain.

In contrast to the *tad* pili, genes encoding for fimbrial proteins such as FimA, FimB, FimP, and FimQ [as described in *B. bifidum* PRL2010 (Turroni et al., 2013)] were not found in all the bifidobacterial strains studied here (Supplementary Table S3; Figure 3). Genes with low homology (30–50%) to *fimA*, *fimP*, and *fimQ* were identified in *B. breve* M-16 V and *B. infantis* R0033, but not in *B. infantis* M-63 (Supplementary Table S3; Figure 3). On the other hand, clear homologs (>70% identity) of these fimbrial-associated genes were identified in *B. bifidum* R0071. The pilus shaft components encoded by *fimA* and *fimP* are polymerized and assembled with pilus tip components (encoded by *fimB* and *fimQ*) by an adjacent class C sortase (srtC). A further class of sortase enzymes [sortase E (srtE)] has also recently been found to be critical for bifidobacterial adhesion (Ishikawa et al., 2021). It is clear, therefore, that specific sortase enzymes may contribute to the adherence phenotypes observed in this study. These sortase-enzymes work by covalently attaching specific sortase-dependent proteins (SDPs) to the cell-wall of gram-positive organisms by recognizing a conserved C-terminal motif. Therefore, to understand if variances in SDPs could account for variances in adherence of the strains studied here, the bifidobacterial proteomes were searched for proteins with a signal peptide at the N-terminus, the srtC (LPXTG) or srtE ([L/I/V][S/A]XTG) recognition motif, followed by a stretch of hydrophobic amino acids and a positively charged C-terminus as previously described (Call and Klaenhammer, 2013; Ishikawa et al., 2021). We found nine such proteins in *B. breve* M-16 V (Supplementary Figure S2; Supplementary Table S4), 13 in *B. infantis* R0033 (Supplementary Figure S2; Supplementary Table S5), and 10 in *B. infantis* M-63 (Supplementary Figure S2; Supplementary Table S6). Analysis of the *B. infantis* strains revealed the presence of fimbrial proteins in R0033 (at BINF_1976 and BINF*_*1977, Supplementary Table S6), which contained the srtC-recognition motif LPXTG and, in the case of BINF*_*1977, a Von Willebrand Factor-type A (VWA) domain associated with binding of extracellular matrix components proteins and host cells (Supplementary Table S5). In contrast, no such srtC-dependent fimbrial proteins were identified in *B. infantis* M-63 (Supplementary Table S6).

In comparison to the lower numbers of SDPs identified in the other bifidobacterial strains, we identified 36 putative sortase-dependent proteins in *B. bifidum* R0071, including seven proteins containing the srtC-recognition motif and 29 proteins containing the srtE-recognition motif (Supplementary Figure S2; Supplementary Table S7). Of these putative srtE-dependent proteins identified in *B. bifidum* R0071, 15 were found to be glycosyl hydrolases (GHs) associated with degradation of HMOs and mucins such as those expressed on the epithelial cell surface, while the predicted srtC-dependent proteins (genetically) corresponded to the fimbrial proteins FimA/ FimP major pilin subunits or FimB/ FimQ minor pilin subunits. In *B. bifidum* R0071 we identified six putative sortase-dependent pilus proteins arranged in three pilin clusters at *BBIF*_1805–1807 (*pil1*: *BBPR*_1820–1822 in PRL2010), *BBIF*_1693–1,695 (*pil2*: *BBPR*_1707–1709) and *BBIF*_0313–0315 (*pil3*: *BBPR*_0282–0284). A recent study has established that the presence of a stretch of polyG influences the on–off switch in the expression of these pili genes (Penno et al., 2022). Therefore, the identified pilin clusters in *B. bifidum* R0071 were searched for the presence of a G-tract up to 500 bp upstream and 200 bp downstream of the predicted gene start in the first gene of the operon. In comparison to PRL2010, which harbours only one G-tract containing pilus gene cluster (Penno et al., 2022), polyG sequences were found to be present in two pilus-specifying gene clusters in *B. bifidum* R0071. A G-tract was identified in the *pil3* gene cluster (*BBIF*_0313–0315) at the position 363939–36394911 (11 base pairs) and in the *pil1* gene cluster (*BBIF*_1805–1807) at the position 2216145 to 2216157 (13 base pairs).

### 3.3 Quantitative proteomic analysis of colonization-related factors

Label-free quantitative proteomic analysis was carried out on the four-strain bifidobacterial mixture after cell exposure and compared to control bacteria that had not been in contact with HT29-MTX cells. Furthermore, the impact of S-HMO pre-treatment on colonization-associated pathways was also assessed by comparing HMO-treated and untreated bifidobacteria following exposure to the human cell line. Preliminary screenings of the adhered cell lysate (which contained both lysed HT29-MTX cells and adhered bacteria) showed substantial masking of bacterial proteins. A total of 2,908 proteins were detected with only 12 proteins matched to bacterial proteins (2 × *B. infantis,* 2 × *B. breve,* and 8 × *B. bifidum*). The remaining 2,896 proteins were matched to human proteins. Since analysis of the adhered cell lysate did not allow for in-depth understanding of the impact of HMO and cell treatments on the bifidobacterial mixture, these samples were eliminated from subsequent analyses. Analysis of bifidobacteria aspirated from the cell apical compartment resulted in the identification of 2,191 bacterial proteins (which covered 37.4% of the proteome of the strains studied here) and 1,385 human proteins. Supplementary Figure S3 shows the distribution of shared or specific bacterial proteins by species (Supplementary Figure S3A) and by treatment (Supplementary Figure S3B). Of the 2,191 bacterial proteins identified, 170 proteins could not be distinguished based on species, with peptides matched to proteins in more than one species (Supplementary Figure S3A). Functional characterization confirmed that these shared proteins were not associated with bacterial adhesion. The remaining proteins detected during label-free quantitative proteomic analysis were matched exclusively to a single proteome in the database, with 393 from *B. bifidum*, 742 from *B. breve* and 886 from *B. infantis*.

**Figure 4:**
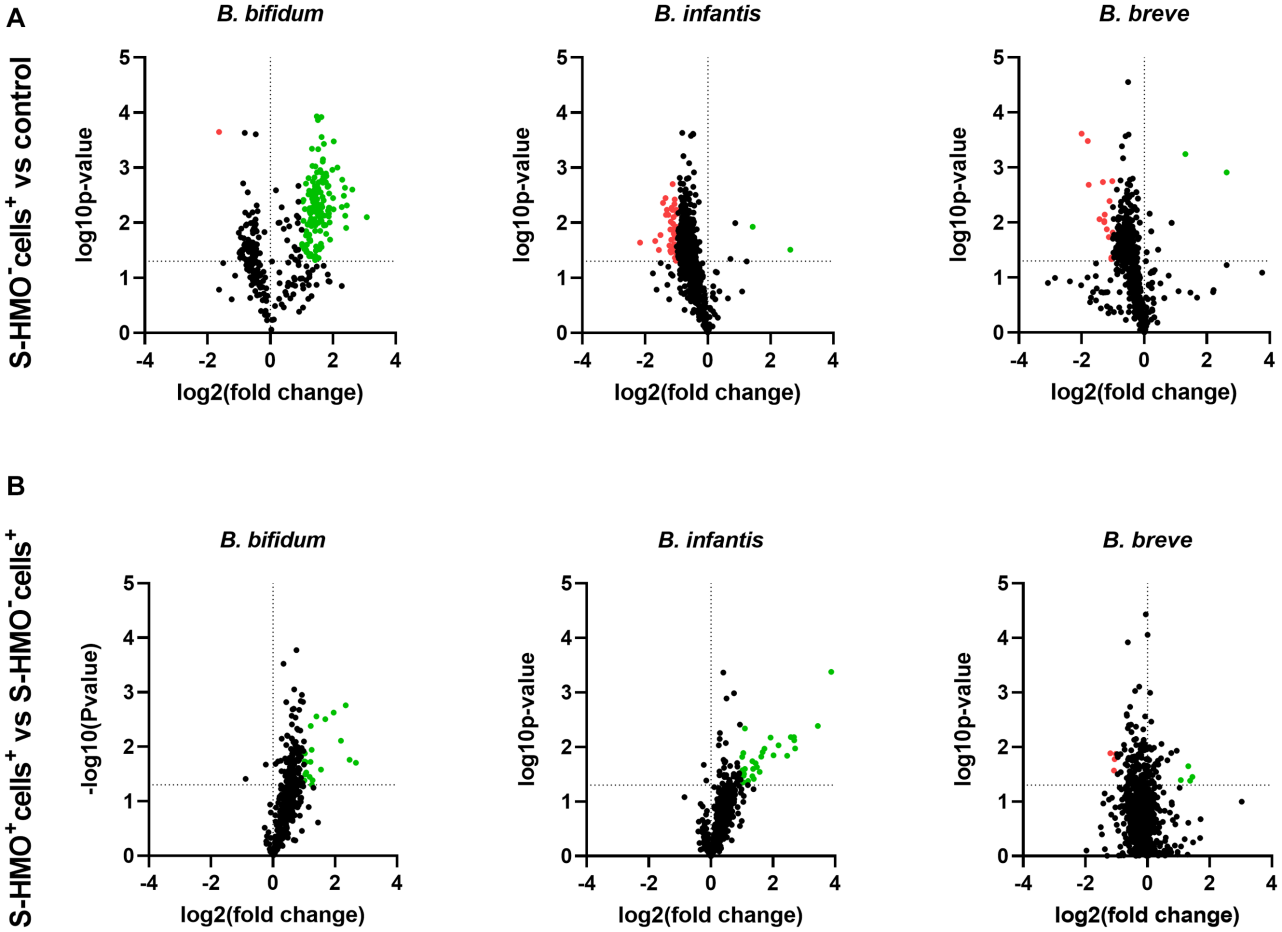
Volcano plots displaying quantitative changes in abundance of *Bifidobacterium bifidum, Bifidobacterium infantis,* and *Bifidobacterium breve* proteins following treatment **(A)** with and without mucus-secreting HT29-MTX cells (S-HMO^−^cells^+^ vs. S-HMO^−^cells^−^) **(B)** with mucus-secreting HT29-MTX cells with and without pre-exposure to S-HMO (S-HMO^+^cells^+^ vs. S-HMO^−^cells^+^) as detected using label-free quantitative proteomics. The horizontal line represents a value of p of 0.05. Proteins with a significant change in abundance [*p* < 0.05 (Student’s *t*-test), fold change ≥2] are highlighted in red to indicate decreased abundance and green to indicate increased abundance.

To assess the influence of epithelial cell exposure on bifidobacterial proteome expression, a 2-fold increase or decrease in the level of protein abundance was used as a cut-off for statistically significant differentially abundant (SSDA) proteins (*p* < 0.05). Following treatment with cells (S-HMO^−^cells^+^), only two *B. infantis* proteins were found in higher abundance when compared to the control group; 30S ribosomal protein S17 (fold increase of 6.37) and sugar kinase (fold increase of 2.74) (Supplementary Table S9). A further two *B. infantis* proteins were uniquely detected after treatment with cells; DNA polymerase III delta subunit and uracil-xanthine transport protein (Supplementary Table S9). Similarly, treatment with cells resulted in increased abundances of only two *B. breve* proteins (30S ribosomal protein S17, and undecaprenyl-phosphate α-*N*-acetylglucosaminephosphotransferase) with a further two proteins uniquely detected after treatment with cells (prolipoprotein diacylglyceryl transferase and a transport protein) (Supplementary Table S10). In contrast, 42 *B. infantis* and 14 *B. breve* proteins decreased in abundance following incubation with HT29-MTX cells (S-HMO^−^cells^+^) (Figure 4A). Qualitative analysis revealed that a further 49 *B. infantis* and 60 *B. breve* proteins were no longer detected after cell-treatment (S-HMO^−^cells^+^). As shown in Figure 4B, pre-treatment with HMO shifted the proteome of these strains, with a number of *B. infantis* proteins, in particular, increasing in abundance. For *B. infantis* it was found that 31 proteins increased in abundance following HMO treatment. Functional characterization, however, revealed that the majority of these upregulated proteins (>90%) were associated with metabolism of HMO, including carbohydrate transporters, glycosyl hydrolases, and bifid shunt enzymes. For *B. breve* it was found that 4 proteins increased in abundance following pre-exposure to HMO (Figure 4B). Functional characterization again revealed that the majority of these proteins (75%) were associated with carbohydrate metabolism.

**Figure 5:**
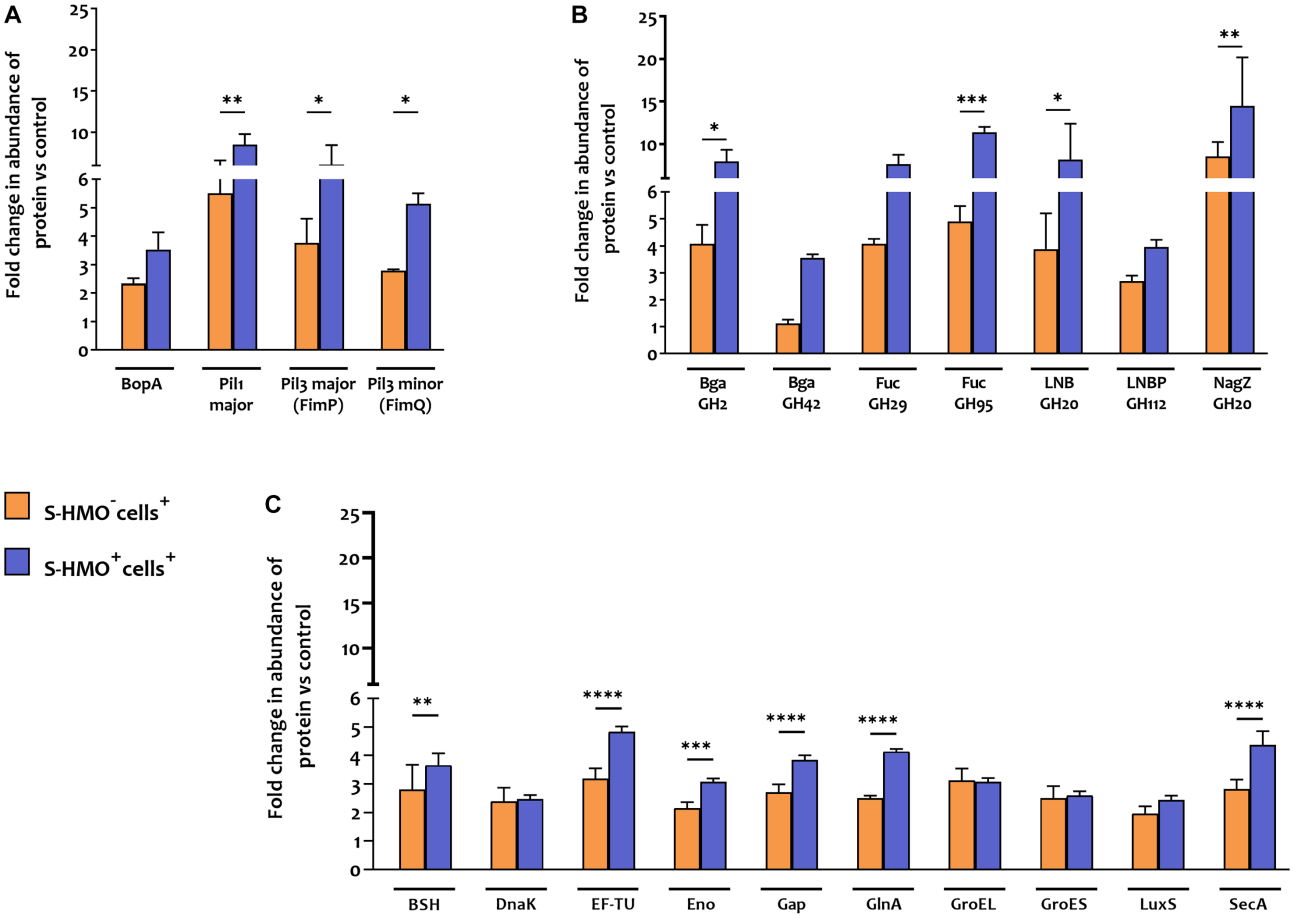
Proteins matched to *Bifidobacterium bifidum* R0071 with potential roles in bifidobacterial colonization that increased in abundance following treatment with cells (± S-HMO). **(A)** Cell surface adhesins **(B)** glycosyl hydrolases **(C)** secondary proteins involved in probiotic persistence. Protein abundance plotted as fold change versus negative control (S-HMO^−^cells^−^, without S-HMO or cell treatment). Analysis was calculated using technical triplicate data from biological triplicate experiments and data are means ± SD. Univariate analysis of variance (ANOVA) and post-hoc Tukey tests were performed to determine the significant differences between the groups (*p < 0.05, **p < 0.01, ***p < 0.001, ****p < 0.0001).

In contrast to *B. infantis* and *B. breve*, only nine *B. bifidum* proteins were detected at lower abundance after cell exposure (S-HMO^−^cells^+^) including 8 proteins detected exclusively in the control group (Figure 4A). On the other hand, incubation of the bifidobacterial mixture with HT29-MTX cells (S-HMO^−^cells^+^) resulted in an increased abundance of 225 *B. bifidum* proteins (including 71 uniquely detected proteins), many of which were further upregulated through pre-treatment with HMO (S-HMO^+^cells^+^) (Figure 4A; Supplementary Table S8). Within this group of upregulated *B. bifidum* proteins, we identified 27 with a potential function in probiotic colonization. The fold changes of these proteins (*vs* control) are summarized in Figure 5 and include cell surface pilus-associated proteins (Figure 5A), glycosyl hydrolases that have been shown to interact with the mucus layer (Figure 5B), and moonlighting proteins associated with bacterial adhesion (Figure 5C). Notably, we observed upregulation of numerous srtC-dependent proteins (as identified in section 3.3) following HT29-MTX exposure. These include Pil3 fimbrial subunits FimP (*BBIF*_0314) and FimQ (*BBIF*_0313) which increased 3.77 fold and 2.79 fold, respectively, following exposure to HT29-MTX cells (HMO^−^cells^+^) (Figure 5A; Supplementary Table S8). These proteins were further upregulated by pre-treatment with HMO (S-HMO^+^cells^+^); with FimP increasing 5.76 fold (vs control) and FimQ increasing 5.13 fold (vs control) (Figure 5A). The Pil1 major pilin (encoded by gene *BBIF*_1806) was also highly upregulated following S-HMO^−^cells^+^ and S-HMO^+^cells^+^ treatment (Figure 5A; Supplementary Table S8).

In addition to SrtC-dependent cell surface appendages, other *B. bifidum* R0071 colonization-related proteins were identified that were upregulated following cell +/− S-HMO treatment (Figure 5C). The majority of these upregulated proteins normally function as housekeeping genes or act as enzymes for glycolysis and are expressed intracellularly. However, these proteins have moonlighting properties and when expressed on the cell surface can facilitate cell adhesion. For example, cell treatment (S-HMO^−^cells^+^) resulted in significant upregulation of chaperone protein DnaK (2.39 fold increase vs. control), chaperonin protein GroES (2.5 fold increase), chaperonin protein GroEL (3.11 fold increase), and S-ribosylhomocysteine lyase LuxS (2.12 fold increase) (Candela et al., 2007; Christiaen et al., 2014; Duranti et al., 2014; Wei et al., 2016; Zhu et al., 2016). Pre-treatment with HMO (S-HMO^+^cells^+^) resulted in significant increases in many moonlighting proteins (Figure 5C). These include elongation factor thermo unstable (EF-Tu), enolase (Eno), and glyceraldehyde phosphate dehydrogenase (Gap) which were upregulated 4.84 fold, 3.07 fold, and 3.84 fold, respectively, following incubation with both cells and HMO (S-HMO^+^cells^+^), which represents a significant increase when compared to treatment with cells alone (S-HMO^−^cells^+^) (Figure 5C; Supplementary Table S8).

Aside from upregulation of cell surface adhesins, we also observed significant upregulation of SrtE-dependent glycosyl hydrolase (GH) proteins in *B. bifidum* R0071 following exposure of the bifidobacterial mixture to mucus-secreting HT29-MTX cells. The cumulative effects of HMO and cell treatment (S-HMO^+^cells^+^) resulted in significant upregulation of numerous SrtE-dependent GH proteins (including β-galactosidases and fucosidases) when compared to treatment with cells alone (S-HMO^−^cells^+^) (Figure 5B).

## 4 Discussion

Our research specifically delved into the adhesive characteristics of four bifidobacterial strains: *Bifidobacterium bifidum* R0071, *Bifidobacterium longum* subsp. *infantis* R0033, *Bifidobacterium breve* M-16 V, and *Bifidobacterium longum* subsp. *infantis* M-63 in the presence of HMO. These strains have garnered considerable attention due to their well-documented health benefits, as substantiated by clinical investigations (Giannetti et al., 2017; Wong et al., 2019; Xiao et al., 2019). For instance, infants fed with a probiotic supplement comprising *B. bifidum* R0071, *B. infantis* R0033, and a *Lactobacillus* strain maintained elevated levels of fecal SIgA after a four-week treatment period (Xiao et al., 2019). *Bifidobacterium breve* M-16 V, another commonly used probiotic in infants, has exhibited promise in safeguarding against conditions such as necrotizing enterocolitis (NEC) and allergic diseases in infants, as extensively reviewed by Wong et al. in 2019 (Wong et al., 2019). Furthermore, a blend containing *B. infantis* M63, along with two other probiotic strains (including M-16 V), demonstrated superior management of functional abdominal pain and a notable enhancement in the quality of life among children with irritable bowel syndrome (IBS) (Giannetti et al., 2017). Notably, our previous research has highlighted the synergistic capabilities of these bifidobacterial strains when it comes to utilizing human milk oligosaccharides (HMOs) as a primary carbohydrate source, resulting in a marked increase in bacterial proliferation (Walsh et al., 2022).

Adherence of probiotic bacteria was evaluated using HT29-MTX human tumorigenic cell lines to mimic bifidobacterial adhesion to intestinal epithelial cells (IECs). The HT29-MTX cell line, which is able to constitutively produce mucin, has more physiologically relevant characteristics when compared to HT29 cells alone due to the mucus layer formation, and has therefore been proposed as a more suitable cell line for studying host–microbe interactions. In agreement with other studies (Kavanaugh et al., 2013; Zhang et al., 2020) through the use of colonisation assays, we confirm that the adhesive potential of bifidobacterial strains can be substantially increased through treatment with milk oligosaccharides. These HMO-induced changes in bifidobacterial adhesion align with findings of Chicklowski et al., where increased adherence of *B. infantis* was observed resulting from exposure to breastmilk-derived HMOs (Chichlowski et al., 2012). However, in contrast to Chicklowski et al., the current study found that HMO-exposed *B. bifidum* had a higher rate of adhesion to intestinal cells compared to *B. infantis* or *B. breve* (Chichlowski et al., 2012). This agrees with previous studies which showed that *B. bifidum* PRL2010 displays higher levels of adhesion to extracellular matrix components (fibronectin, plasminogen, and laminin) when compared with *B. breve* and *B. infantis* (Turroni et al., 2013). Notably, proteomic analysis of the bifidobacterial combination revealed that many proteins within the *B. infantis* and *B. breve* proteome decreased in abundance following exposure to intestinal cells. This is likely a result of complex and dynamic interactions between the bacteria and host cells. These interactions can trigger various responses, including adaption to host immune responses, regulatory changes, and resource allocation, which collectively influence the protein expression profile of these bacteria during the assay.

Of note, we observed higher levels of adhesion in HMO-exposed *B. infantis* R0033 when compared to its species counterpart *B. infantis* M-63. Our findings verify that bacterial adhesion to intestinal epithelial cells is not only species-specific but also strain-specific. Interestingly, we have previously highlighted the strain-level differences between M-63 and R0033, with M-63 demonstrating superior growth on breastmilk-derived HMO (Walsh et al., 2022). It may be important, therefore, when assessing interactions of HMO and probiotics, to consider the growth phenotype but also the adherent phenotype resulting from a strain’s exposure to HMO. Notably, findings from adhesion assays using a combination of all four strains have indicated that co-operation between strains may expand HMO acquisition capabilities to enhance gut colonization activity of strains. qPCR analysis of the bifidobacterial consortia revealed that, irrespective of the carbon source, *B. bifidum* R0071 occupied the largest proportion of bacteria adhering to HT29-MTX cells. Interestingly, recent studies have indicated that strains *of B. bifidum* adhere more strongly to infant mucus than adult mucus, while more adult-associated species such as *B. lactis* and *L. rhamnosus* adhere more strongly to adult mucus than to infant mucus. In general, the adhesion of *B. bifidum in vitro* is exceptionally strong among probiotics (Shibahara-Sone et al., 2016) and therefore the high levels of adherence observed in this study may be expected. Such characteristics might arise from the vast array of sortase-dependent proteins specific to *B. bifidum* as described in a recent study (Ishikawa et al., 2021).

The survey of the bacterial genomes for colonisation-related factors revealed a number of components that bacteria can potentially interact with to adhere to mucus and epithelial surface glycans, e.g., outer-membrane proteins, adhesins, capsules and appendages such as pili, flagella, and fimbriae (Paone and Cani, 2020). Assembly of pilus-like appendages on the bacterial surface is thought to be pivotal for bifidobacterial gut colonization. In recent years, pilus encoding-gene clusters have been identified in many infant-derived bifidobacteria, most notably a type IVb tight adherence (Tad) pilus-encoding gene cluster in *B. breve* UCC2003 (O'Connell Motherway et al., 2011) and sortase-dependent pilus gene clusters in *B. bifidum* PRL2010 (Turroni et al., 2013). The fact that genes with homology to the UCC2003 *tad* locus were found in all our strains aligns with studies from O’Connell Motherway et al., where the authors found that the tad pilus-encoding locus was highly conserved across the bifidobacterial genera (O'Connell Motherway et al., 2011). That study suggested that such a phenomenon supports the notion of a ubiquitous pili-mediated host colonization and persistence mechanism for bifidobacteria (O'Connell Motherway et al., 2011, 2019).

In addition to tad pili, membrane-bound transpeptidase enzymes known as “sortases” have been found to play a critical role in facilitating bacterium-host cell crosstalk throughout the bifidobacterial genera. In fact, a recent study from Ishikawa et al. (2021) found that non-adhesive strains belonging to the *B. bifidum* species lacked a functional class E (housekeeping) sortase (Ishikawa et al., 2021). Class E sortases (srtE) work by anchoring proteins to the cell surface, while class C sortases (srtC) are responsible for constructing pilus polymers. Sortase-mediated surface structures are responsible, therefore, for cell attachment and nutrient uptake, amongst other functions. The small number of putative srtC-dependent clusters associated with pilus expression identified in *B. infantis* strains is in line with a previous study in which pilus-like structures were shown to be rare if at all present in *B. longum* subsp. *infantis* ATCC15697 (Foroni et al., 2011). Since pilus-like structures in *B. infantis* are rare, the srtC-dependent fimbrial units (containing VWA-domains) identified in R0033 may be notable. VWA-domains have been found in pilus proteins that bind to extracellular matrix components and host cells (Whittaker and Hynes, 2002). Follow-up studies are required to elucidate whether the expression of these fimbriae proteins in R0033 could account for its superior adherence over its *B. infantis* counterpart M-63. In contrast to the *B. infantis* and *B. breve* strains studied here, numerous srtC-dependent fimbrial proteins were identified in *B. bifidum* R0071. As described for *B. bifidum* PRL2010, the genome of *B. bifidum* R0071 encompasses three different loci (Pil1, Pil2, and Pil3) encoding the predicted biosynthetic machinery for the production of sortase-dependent pili (Turroni et al., 2013). Proteomic analysis of the bifidobacterial combination following exposure to intestinal cells revealed substantial upregulation of srtC-dependent pilin proteins within two pilus-specifying clusters (Pil1 and Pil3) but not Pil2. Recent studies have highlighted that the presence of a stretch of polyG influences the on–off switch in the expression of these pili genes. We confirm that polyG sequences were found downstream of the predicted gene start in Pil1 and Pil3, but not Pil2. The identification of polyG sequences is noteworthy, as productive replication slippage at G-tract sequences may be an important mediator of sortase-dependent pili expression and subsequent colonization of bifidobacterial strains within the gastrointestinal tract (Penno et al., 2022). Importantly, significant upregulation of the pilus proteins within the *B. bifidum* Pil1 and Pil3 clusters was observed following exposure of the bifidobacteria to HMO. The HMO-induced upregulation of srtC-dependent pilins aligns with findings from Foroni et al. where high production of pilus-like structure was observed following propagation of *B. bifidum* PRL2010 on fructooligosaccharides (Foroni et al., 2011). It is clear therefore, that expression of sortase-dependent pili by *B. bifidum* can be influenced by the presence of complex carbohydrates and may account for the increased adherence of this strain following exposure to S-HMO.

A somewhat less explored category of proteins known as “moonlighting proteins” (Huberts and van der Klei, 2010) may also play a critical role in bacterial adhesion. Most of these proteins typically serve as housekeeping genes or function as enzymes in glycolysis and are expressed intracellularly. Nevertheless, these proteins have been found to be multifunctional and, when expressed on the cell surface, have been reported to facilitate cell adhesion. In this current study, moonlighting proteins with primary functions as chaperone proteins such as DnaK and GroEL were more abundant in the *B. bifidum* R0071 proteome following co-cultivation with IECs. This aligns with previous research in *Lactobacillus johnsonii* (Bergonzelli et al., 2006) and *Bifidobacterium animalis* (Candela et al., 2010) which demonstrated a role for GroEL and DnaK in probiotic binding to mucins and plasminogen. However, in contrast to Kavanaugh et al., we found that exposure of bifidobacteria to HMO did not influence the expression of DnaK or GroEL (Kavanaugh et al., 2013), although these discrepancies could be accounted for differences in strains (multistrain vs. single strain of *B. infantis*), HMOs (breastmilk-derived HMO vs. combination of 3′-SL and 6′-SL), or analytical methods (transcriptomic vs. proteomic profiling). However, we identified numerous moonlighting proteins that serve as adhesins for bifidobacteria in higher abundance in the *B. bifidum* R0071 proteome following co-cultivation with HT29-MTX cells which were further upregulated following pre-exposure to HMO. These include enzymes typically associated with glycolysis and the bifid shunt pathway such as transaldolase (Tal), enolase (Eno), and glyceraldehyde-3-phosphate dehydrogenase (Gap) (Pokusaeva et al., 2011). This aligns with previous studies which have identified mucin-binding properties for transaldolase in *B. bifidum* (González-Rodríguez et al., 2012) and plasminogen-binding properties for enolase in *B. longum*, *B. bifidum*, *B. lactis*, and *B. breve* strains (Candela et al., 2007, 2009; Wei et al., 2014). Similar plasminogen binding capabilities have also been demonstrated for EF-Tu (Candela et al., 2007), which is another moonlighting protein which may facilitate cell adhesion and which was found at significantly higher abundances the *B. bifidum* R0071 proteome following IEC exposure and HMO pre-treatment. Overall, the proteomic profiling of moonlighting proteins outlined in this study indicates that bifidobacterial strains may be able to sense the conditions of their intestinal environment to detect receptors on IECs and respond by enhancing expression of adhesive molecules on the bacterial cell surface.

The mucus layer not only supplies attachment sites to commensal bacteria, it also serves as a nutrient source for organisms in the gut microbiota (Hansson, 2020; Paone and Cani, 2020). The turnover of the intestinal mucus layer involves mucus synthesis, secretion and degradation, and represents a delicate process that needs to be regulated and balanced to ensure that mucus maintains an optimal protective function (Schneider et al., 2018). Recent studies have found that mucin adhesion is closely linked to mucin utilization (Ishikawa et al., 2021), with researchers highlighting that the class E housekeeping sortase is a critical component influencing adhesion of *B. bifidum* strains (Ishikawa et al., 2021). This aligns with the identification of SrtE-dependent glycosyl hydrolase proteins in R0071 following exposure the HT29-MTX cells and/ or HMO. Microbes most adept at mucin glycan degradation often encode sialidases and fucosidases to remove terminal structures which allows for greater accessibility to the extended core structures. The monosaccharides released by the action of these enzymes may be utilized by the bacteria themselves or released into the environment for scavenging bacteria (Luo et al., 2021). Proteomic analysis revealed significant upregulation of *B. bifidum* fucosidases belonging to GH29 and GH95 families, which hydrolyse Fucα1–2Gal and Fucα1–3/4Gal linkages, respectively. A SrtE-dependent GH33 exo-α-sialidase was detected that matched to *B. bifidum* R0071 which was found exclusively in bacteria exposed to mucus secreting intestinal cells. The fact that there was significant upregulation of fucosidases enzymes and an exo-α-sialidase in R0071 following incubation with IECs is notable. Studies have shown that the fucose released from mucin glycans by *B. bifidum* enables growth of *Eubacterium hallii*, a commensal species that produces butyrate and propionate from fermentation metabolites (Bunesova et al., 2018). Sialic acid residues are another highly sought-after nutrient source that are found terminating mucin glycan chains. To access this glycan residue intestinal bacteria express GH33 sialidases, which cleave terminal sialic acid residues. Previous studies have highlighted that cleavage of sialic acid residues from mucin oligosaccharides, catalyzed by *B. bifidum* GH33 sialidases, can support growth of *B. breve* UCC2003 (Egan et al., 2014). We hypothesize therefore that HMO-enhanced colonization of *B. bifidum* could also benefit other gut symbionts through mucin cross-feeding activities.

## 5 Conclusion

Overall, we confirm that adherence of bifidobacterial strains to intestinal epithelial cells is species- and strain-specific. Our results suggest that HMO, a prominent component of human breast milk, can promote interaction of infant-derived bifidobacteria and intestinal cells. In particular, we show that the presence of HMOs significantly improves the adhesion ability of a mixed community of commercial bifidobacterial strains. The adherence of bifidobacteria to intestinal epithelial cells is essential for their colonization, their ability to support a healthy gut environment, and their potential to confer various health benefits to the host. Recent studies have shown that certain bifidobacterial species, including *B. infantis*, are in danger of becoming extinct due to a multitude of factors including changes in breastfeeding habits in certain countries, C-sections deliveries, and antibiotic usage (Taft et al., 2022). Therefore, it has never been more important to explore a means to increase the colonization of beneficial bacteria such as bifidobacteria in the gut. Specifically, we show that carbohydrate sources are important environmental factors influencing adhesin expression levels particularly in *B. bifidum*, which may have knock-on effects positively influencing trophic interactions and social behavior among bifidobacterial species and between other intestinal microbes as has been shown previously for this species (Egan et al., 2014; Bunesova et al., 2018). We confirm that assembly and localization of surface proteins, including SrtC-dependent pili and moonlighting proteins, are key factors influencing *B. bifidum* adhesion. Our findings also verify that the class E housekeeping sortase may be a critical component influencing adhesion of *B. bifidum* strains by increasing bifidobacterial interaction with the mucin layer. Although *in vitro* experiments are key to understand the mechanisms of adhesion and select probiotic candidates with potential to adhere *in vivo*, it is difficult to extrapolate these results to the human intestinal tract where other factors such as peristaltic movements, host immune system, diet and genetics, or competition with resident microbiota could interfere with attachment. It is clear, therefore, that further clinical studies with probiotics, which incorporate specific combinations of probiotic consortia and HMOs, will be critical in understanding how the findings outlined here translate to the *in vivo* situation. Nevertheless, this study sheds light on the future use of oligosaccharides and bifidobacteria for nutritional intervention or clinical applications.

## Data availability statement

Genome data presented in this study for R0033 and R0071 is deposited in the Bacterial and Viral Bioinformatics Resource Center (BV-BRC) using the following links: R0033: https://www.bv-brc.org/view/Genome/1678.111, R0071: https://www.bv-brc.org/view/Genome/1678.107. Genome data for M-16V and M-63 is deposited in the NCBI repository, accession number OR940152-64.

## Author contributions

CW designed the experiments, performed the experiments and data analysis, prepared the original draft, and contributed to writing—reviewing and editing. RO contributed to proteomic analysis and writing—reviewing and editing. FB contributed to sequence assembly and annotation, writing—reviewing and editing. JL contributed to writing—reviewing and editing. DS and RH contributed to writing—reviewing and editing and supervision. All authors contributed to the article and approved the submitted version.

## Footnotes

^1^ https://github.com/PacificBiosciences/SMRT-Analysis

^2^ http://prodigal.ornl.gov

^3^ http://ftp.ncbi.nih.gov/blast/db/

^4^ http://pfam.sanger.ac.uk

^5^ http://www.sanger.ac.uk/resources/soft-ware/artemis/

^6^ http://bioinformatics.psb.ugent.be/webtools/Venn/
