# Supplementary material for: HMO-primed bifidobacteria exhibit enhanced ability to adhere to intestinal epithelial cells
Source: Front Microbiol. 2023 Dec 15;14:1232173. doi: 10.3389/fmicb.2023.1232173 (PMC10757668; doi:10.3389/fmicb.2023.1232173)
Supplement: Supplementary file 1 [file Data_Sheet_1.PDF]

Supplementary Table 1: Fold change in adherence levels of strain(s) versus *B. bifidum* R0071

	***B. bifidum* R0071**	***B. infantis* R0033**	***B .breve* M-16V**	***B. infantis* M-63**	**4Bif**
**Control**	1.00 ± 0.087[a]	0.89 ± 0.169[a]	0.62 ± 0.077[b]	0.79 ± 0.166[b]	1.15 ± 0.107[a]
**S-HMO**	1.00 ± 0.106[a]	0.89 ± 0.112[b]	0.57 ± 0.118[c]	0.76 ± 0.131[d]	1.65 ± 0.252[e]
**2'-FL**	1.00 ± 0.105[a]	1.05 ± 0.092[a]	0.66 ± 0.076[b]	0.99 ± 0.132[a]	1.31 ± 0.261[c]

Fold change calculated as (percent adherence [vs original inoculum] of strain)/ (percent adherence [vs original inoculum] of *B. bifidum* R0071)
Values within a row with common letter= p > 0.05, calculated using Tukey's multiple comparisons test

Supplementary Table 2: Species concentration and significant differences between strains within treatment groups following 16S qPCR analysis of adhered bifidobacterial mixture (4Bif)

	**x 10^5 bifidobacteria/mL**					
**Treatment group ↓**	***B. bifidum***		***B. breve***		***B.infantis***	
	Mean	StDev	Mean	StDev	Mean	StDev
**Control**	2.21[a]	0.87	0.45[a]	0.10	1.51[a]	0.64
**HMO**	5.64[a]	1.50	1.14[b]	0.12	3.13[b]	2.21
**2'FL**	2.74[a]	0.30	0.68[a]	0.07	1.82[a]	0.64

Values within a row with common letter= p > 0.05, calculated using Tukey's multiple comparisons test

Supplementary Table 3: Bifidobacterial genomes matched against genes associated with probiotic survival and colonisation

Gene name	Protein encoded	Strain*	Ref	% identity** *B. bifidum* R0071	*B. breve* M16V	*B. infantis* R0033	*B. infantis* M63
bl0155	Large transmembrane protein	*B. longum* VMKB44	(Shkoporov et al., 2008)	55.43	80.66	94.30	94.22
bl0675	Fimbrial subunit FimA	*B. longum* NCC2705	(Foroni et al., 2011)	43.95	26.99	27.90	0.00
bopA	Putative cell surface lipoprotein	*B. bifidum*PRL2010	(Guglielmetti et al., 2008)	98.82	46.09	43.83	44.01
bsh	Bile salt hydrolase	*B. longum* SBT2928	(Tanaka et al., 2000)	90.764	99.369	99.054	98.738
copA	Copper transporting atpase	*L. plantarum* NZ7109b	(Bron et al., 2007)	36.02	37.93	33.63	33.43
ctr	(Na+) symporter	*B. longum* NCC2705	(Gueimonde et al., 2009)	0.00	92.118	94.842	94.842
dltA	D-alanine--D-alanyl carrier protein	*L. reuteri* 100- 23	(Walter et al., 2007)	0.00	23.35	32.24	23.62
ebp	Elastin-binding protein	*B. bifidum* PRL2010	(Turroni et al., 2013)	96.52	42.54	42.50	42.00
ef-Tu	Elongation factor	*L. johnsonii* NCC533	(Granato et al. 2004)	0.00	66.08	66.332	66.332
esat-6	ESAT-6-like protein	*B. bifidum* PRL2010	(Turroni et al., 2013)	100.00	83.16	86.46	86.46
fimA	Pil2 major pilin subunit	*B. bifidum* PRL2010	(Turroni et al., 2013)	99.46	30.00	31.17	0.00
fimB	Pil2 minor pilin subunit	*B. bifidum* PRL2010	(Turroni et al., 2013)	99.45	0.00	0.00	0.00
fimM	Major pilin subunit	*B. longum* BBMN68	(Xiong et al. 2020)	30.66	26.42	30.49	0.00
fimP	Pil3 major pilin subunit	*B. bifidum* PRL2010	(Turroni et al., 2013)	95.32	33.57	32.40	0.00

Supplementary Table 3: Bifidobacterial genomes matched against genes associated with probiotic survival and colonisation

Gene name	Protein encoded	Strain*	Ref	% identity**			
				***B. bifidum* R0071**	***B. breve* M16V**	***B. infantis* R0033**	***B. infantis* M63**
fimQ	Pil3 minor pilin subunit	*B. bifidum* PRL2010	(Turroni et al., 2013)	98.21	37.33	31.52	0.00
glgA	Glycogen synthase	*L. acidophilus* NCK1909	(Goh and Klaenhammer, 2014)	24.13	24.30	22.43	22.22
luxS	S-ribosylhomocysteine lyase	*B. breve* UCC2003	(Christiaen et al., 2014)	90.24	98.78	98.17	97.56
msrB	Peptide methionine sulfoxide reductase MsrB	*L. reuteri* 100- 23C	(Walter et al., 2005)	61.19	60.45	60.45	60.45
nreB	Two-component system, histidine kinase	*L. reuteri* 100- 23C	(Frese et al., 2011, Frese et al., 2013)	26.24	22.18	24.47	24.34
rfbP	Undecaprenyl-phosphate galactosephosphotransferase	*B. breve* UCC2003	(Fanning et al., 2012)	76.99	98.79	98.94	74.56
secA2	SecA2 translocase	*L. reuteri* 100- 23C	(Frese et al., 2011, Frese et al., 2013)	35.17	35.01	34.93	34.93
secY	Secy2, involved in secA2 transport system	*L. reuteri* 100- 23C	(Frese et al., 2011, Frese et al., 2013)	25.27	24.67	24.40	24.40
spaCc	Secreted LPXTG-like pilins	*L. rhamnosus* GG (LGG)	(Kankainen et al., 2009)	32.80	25.44	31.66	0.00
srtA	Pil3 sortase	*B. bifidum* PRL2010	(Turroni et al., 2013)	98.47	70.40	62.53	61.16
srtA	Sortase-like protein	*B. longum* NCC2705	(Foroni et al., 2011)	85.627	38.369	40	41.87
srtE	Sortase family protein	*B. bifidum* PRL2010	(Ishikawa et al., 2021)	99.27	67.05	58.02	58.96
tadA	Type II/ IV secretion system protein	*B. breve* UCC2003	(O'Connell Motherway et al., 2011)	66.97	99.41	73.25	73.25

Supplementary Table 3: Bifidobacterial genomes matched against genes associated with probiotic survival and colonisation

Gene name	Protein encoded	Strain*	Ref	% identity** B. bifidum R0071	B. breve M16V	B. infantis R0033	B. infantis M63
tadB	TadB-like protein	*B. breve* UCC2003	(O'Connell Motherway et al., 2011)	42.466	98.649	67.808	67.123
tadC	Lipoprotein lipid attachment site, TadC-like	*B. breve* UCC2003	(O'Connell Motherway et al., 2011)	55.814	98.624	54.187	54.187
tadE	Pilus assembly protein TadE	*B. breve* UCC2003	(O'Connell Motherway et al., 2011)	39.13	100.00	0.00	0.00
tadF	Pilus assembly protein TadF	*B. breve* UCC2003	(O'Connell Motherway et al., 2011)	41.51	100.00	57.41	57.41
tadV	Type IV secretion peptidase	*B. breve* UCC2003	(O'Connell Motherway et al., 2011)	55.4	98.73	90.28	90.28
tagB	Teichoic acid biosynthesis protein	*B. bifidum* PRL2010	(Turroni et al., 2013)	99.52	0.00	0.00	0.00
tal	Aldolase	*B. bifidum* A8	(González-Rodríguez et al., 2012)	98.00	97.00	95.00	95.00
tlyC	Hemolysin-like protein	*B. longum* BBMN68	(Liu et al., 2014)	69.231	89.474	95.833	98.958
ureC	Urease subunit α	*L. reuteri* 100-23C	(Wilson et al., 2014)	0.00	0.00	55.71	55.71

*Strain column refers to strains in which colonization feature was identified

**Percent identity based on BLAST search using e-value cutoff of 1e-5 and query cover cutoff of 50%

Table formatted with graded colour scale based on percent identity in accordance with Figure 3 (higher % identities correspond to darker orange colours)

BRON, P., MEIJER, M., BONGERS, R., DE VOS, W. & KLEEREBEZEM, M. 2007. Dynamics of competitive population abundance of Lactobacillus plantarum ivi gene mutants in faecal samples after passage through the gastrointestinal tract of mice. *Journal of applied microbiology,* 103**,** 1424-1434.

CALL, E. K., GOH, Y. J., SELLE, K., KLAENHAMMER, T. R. & O'FLAHERTY, S. 2015. Sortase-deficient lactobacilli: effect on immunomodulation and gut retention. *Microbiology,* 161**,** 311.

CHRISTIAEN, S. E. A., O'CONNELL MOTHERWAY, M., BOTTACINI, F., LANIGAN, N., CASEY, P. G., HUYS, G., NELIS, H. J., VAN SINDEREN, D. & COENYE, T. 2014. Autoinducer-2 Plays a Crucial Role in Gut Colonization and Probiotic Functionality of Bifidobacterium breve UCC2003. *PLOS ONE,* 9**,** e98111.

FANNING, S., HALL, L. J., CRONIN, M., ZOMER, A., MACSHARRY, J., GOULDING, D., MOTHERWAY, M. O., SHANAHAN, F., NALLY, K., DOUGAN, G. & VAN SINDEREN, D. 2012. Bifidobacterial surface-exopolysaccharide facilitates commensal-host interaction through immune modulation and pathogen protection. *Proc Natl Acad Sci U S A,* 109**,** 2108-13.

FORONI, E., SERAFINI, F., AMIDANI, D., TURRONI, F., HE, F., BOTTACINI, F., O'CONNELL MOTHERWAY, M., VIAPPIANI, A., ZHANG, Z. & RIVETTI, C. Genetic analysis and morphological identification of pilus-like structures in members of the genus Bifidobacterium. Microbial Cell Factories, 2011. BioMed Central, 1-13.

FRESE, S. A., BENSON, A. K., TANNOCK, G. W., LOACH, D. M., KIM, J., ZHANG, M., OH, P. L., HENG, N. C., PATIL, P. B. & JUGE, N. 2011. The evolution of host specialization in the vertebrate gut symbiont Lactobacillus reuteri. *PLoS genetics,* 7**,** e1001314.

FRESE, S. A., MACKENZIE, D. A., PETERSON, D. A., SCHMALTZ, R., FANGMAN, T., ZHOU, Y., ZHANG, C., BENSON, A. K., CODY, L. A. & MULHOLLAND, F. 2013. Molecular characterization of host-specific biofilm formation in a vertebrate gut symbiont. *PLoS genetics,* 9**,** e1004057.

GANESH, B. P., HALL, A., AYYASWAMY, S., NELSON, J. W., FULTZ, R., MAJOR, A., HAAG, A., ESPARZA, M., LUGO, M. & VENABLE, S. 2018. Diacylglycerol kinase synthesized by commensal Lactobacillus reuteri diminishes protein kinase C phosphorylation and histamine-mediated signaling in the mammalian intestinal epithelium. *Mucosal immunology,* 11**,** 380-393.

GOH, Y. J. & KLAENHAMMER, T. R. 2014. Insights into glycogen metabolism in Lactobacillus acidophilus: impact on carbohydrate metabolism, stress tolerance and gut retention. *Microbial cell factories,* 13**,** 1-12.

GONZÁLEZ-RODRÍGUEZ, I., SÁNCHEZ, B., RUIZ, L., TURRONI, F., VENTURA, M., RUAS-MADIEDO, P., GUEIMONDE, M. & MARGOLLES, A. 2012. Role of extracellular transaldolase from Bifidobacterium bifidum in mucin adhesion and aggregation. *Applied and environmental microbiology,* 78**,** 3992-3998.

GUEIMONDE, M., GARRIGUES, C., VAN SINDEREN, D., DE LOS REYES-GAVILÁN, C. G. & MARGOLLES, A. 2009. Bile-inducible efflux transporter from Bifidobacterium longum NCC2705, conferring bile resistance. *Appl Environ Microbiol,* 75**,** 3153-60.

GUGLIELMETTI, S., TAMAGNINI, I., MORA, D., MINUZZO, M., SCARAFONI, A., ARIOLI, S., HELLMAN, J., KARP, M. & PARINI, C. 2008. Implication of an outer surface lipoprotein in adhesion of Bifidobacterium bifidum to Caco-2 cells. Am Soc Microbiol.

ISHIKAWA, E., YAMADA, T., YAMAJI, K., SERATA, M., FUJII, D., UMESAKI, Y., TSUJI, H., NOMOTO, K., ITO, M., OKADA, N., NAGAOKA, M. & GOMI, A. 2021. Critical roles of a housekeeping sortase of probiotic Bifidobacterium bifidum in bacterium–host cell crosstalk. *iScience,* 24**,** 103363.

KANKAINEN, M., PAULIN, L., TYNKKYNEN, S., VON OSSOWSKI, I., REUNANEN, J., PARTANEN, P., SATOKARI, R., VESTERLUND, S., HENDRICKX, A. P. & LEBEER, S. 2009. Comparative genomic analysis of Lactobacillus rhamnosus GG reveals pili containing a human-mucus binding protein. *Proceedings of the National Academy of Sciences,* 106**,** 17193-17198.

LIU, Y., AN, H., ZHANG, J., ZHOU, H., REN, F. & HAO, Y. 2014. Functional role of tlyC1 encoding a hemolysin-like protein from Bifidobacterium longum BBMN68 in bile tolerance. *FEMS Microbiol Lett,* 360**,** 167-73.

O'CONNELL MOTHERWAY, M., ZOMER, A., LEAHY, S. C., REUNANEN, J., BOTTACINI, F., CLAESSON, M. J., O'BRIEN, F., FLYNN, K., CASEY, P. G., MORENO MUNOZ, J. A., KEARNEY, B., HOUSTON, A. M., O'MAHONY, C., HIGGINS, D. G., SHANAHAN, F., PALVA, A., DE VOS, W. M., FITZGERALD, G. F., VENTURA, M., O'TOOLE, P. W. & VAN SINDEREN, D. 2011. Functional genome analysis of <i>Bifidobacterium breve</i> UCC2003 reveals type IVb tight adherence (Tad) pili as an essential and conserved host-colonization factor. *Proceedings of the National Academy of Sciences,* 108**,** 11217-11222.

SHKOPOROV, A. N., KHOKHLOVA, E. V., KAFARSKAIA, L. I., PAVLOV, K. A., SMEIANOV, V. V., STEELE, J. L. & EFIMOV, B. A. 2008. Search for protein adhesin gene in Bifidobacterium longum genome using surface phage display technology. *Bull Exp Biol Med,* 146**,** 782-5.

SIMS, I. M., FRESE, S. A., WALTER, J., LOACH, D., WILSON, M., APPLEYARD, K., EASON, J., LIVINGSTON, M., BAIRD, M. & COOK, G. 2011. Structure and functions of exopolysaccharide produced by gut commensal Lactobacillus reuteri 100-23. *The ISME journal,* 5**,** 1115-1124.

TANAKA, H., HASHIBA, H., KOK, J. & MIERAU, I. 2000. Bile salt hydrolase of Bifidobacterium longum—biochemical and genetic characterization. *Applied and environmental microbiology,* 66**,** 2502-2512.

TURRONI, F., SERAFINI, F., FORONI, E., DURANTI, S., O'CONNELL MOTHERWAY, M., TAVERNITI, V., MANGIFESTA, M., MILANI, C., VIAPPIANI, A., ROVERSI, T., SÁNCHEZ, B., SANTONI, A., GIOIOSA, L., FERRARINI, A., DELLEDONNE, M., MARGOLLES, A., PIAZZA, L., PALANZA, P., BOLCHI, A., GUGLIELMETTI, S., VAN SINDEREN, D. & VENTURA, M. 2013. Role of sortase-dependent pili of <i>Bifidobacterium bifidum</i> PRL2010 in modulating bacterium–host interactions. *Proceedings of the National Academy of Sciences,* 110**,** 11151-11156.

WALTER, J., CHAGNAUD, P., TANNOCK, G. W., LOACH, D. M., DAL BELLO, F., JENKINSON, H. F., HAMMES, W. P. & HERTEL, C. 2005. A high-molecular-mass surface protein (Lsp) and methionine sulfoxide reductase B (MsrB) contribute to the ecological performance of Lactobacillus reuteri in the murine gut. *Applied and environmental microbiology,* 71**,** 979-986.

WALTER, J., LOACH, D. M., ALQUMBER, M., ROCKEL, C., HERMANN, C., PFITZENMAIER, M. & TANNOCK, G. W. 2007. D-alanyl ester depletion of teichoic acids in Lactobacillus reuteri 100-23 results in impaired colonization of the mouse gastrointestinal tract. *Environmental microbiology,* 9**,** 1750-1760.

WALTER, J., SCHWAB, C., LOACH, D. M., GÄNZLE, M. G. & TANNOCK, G. W. 2008. Glucosyltransferase A (GtfA) and inulosucrase (Inu) of Lactobacillus reuteri TMW1. 106 contribute to cell aggregation, in vitro biofilm formation, and colonization of the mouse gastrointestinal tract. *Microbiology,* 154**,** 72-80.

WILSON, C. M., LOACH, D., LAWLEY, B., BELL, T., SIMS, I. M., O'TOOLE, P. W., ZOMER, A. & TANNOCK, G. W. 2014. Lactobacillus reuteri 100-23 modulates urea hydrolysis in the murine stomach. *Applied and environmental microbiology,* 80**,** 6104-6113.

Supplementary Figure 1

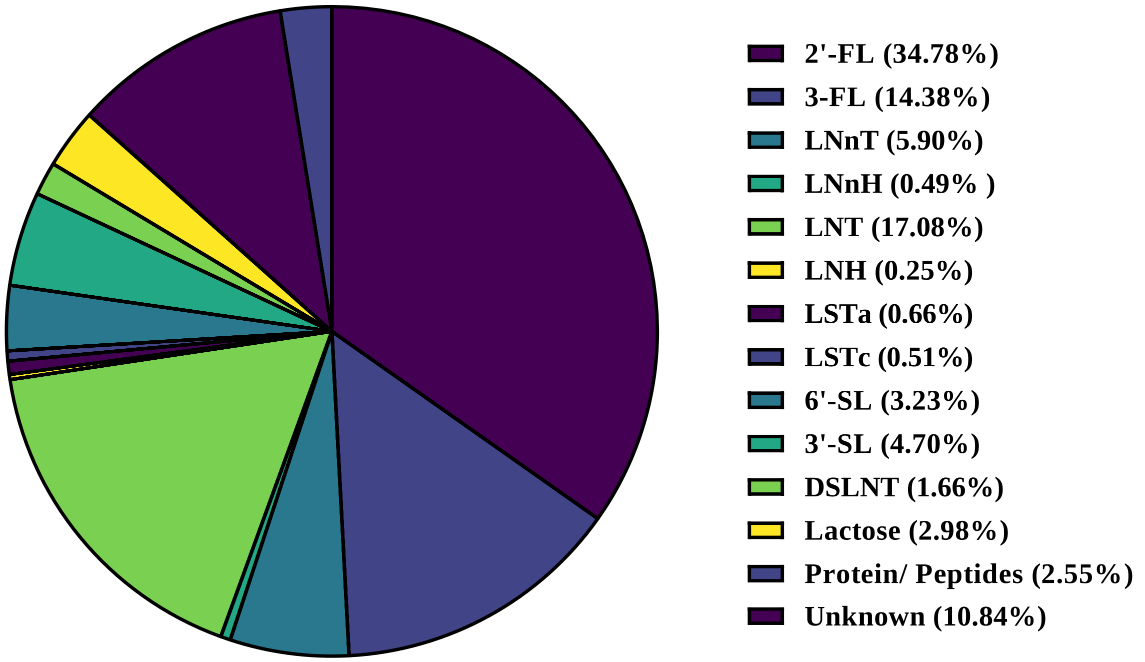

Figure S1: Pie chart showing the relative abundance of the major oligosaccharides in breastmilk-derived HMO which were detected via HPAEC-PAD analysis by comparing to the following external HMO standards: lactose, 3-fucosyllactose (3-FL), 2’-fucosyllactose (2'-FL), lacto-N-neotetraose (LNnT), lacto-N-neohexaose (LNnH), lacto-N-tetraose (LNT), lacto-N-hexaose (LNH), sialyllacto-N-tetraose a (LSTa), sialyllacto-N-tetraose a (LSTc), 6’-sialyllactose (6'-SL), 3’-sialyllactose (3'-SL), disialyllacto-N-tetraose (DSLNT). Data represents average concentrations of technical duplicate data from biological triplicate experiments

Supplementary Figure 2:

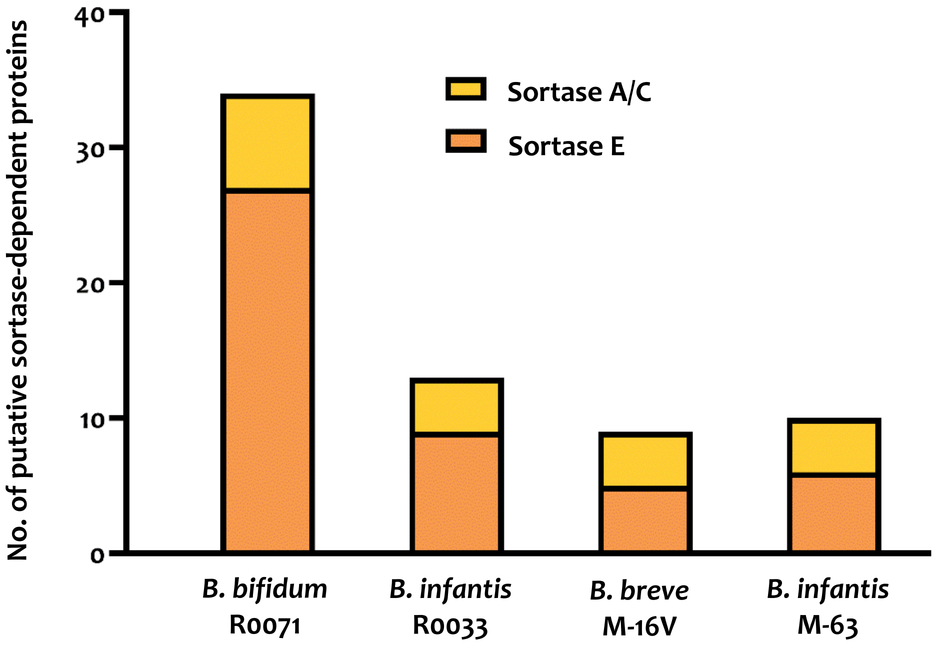

Figure S2: Numbers of putative sortase A/C (yellow) and sortase E (orange)- dependent proteins harboured by bifidobacterial strains *Bifidobacterium bifidum* R0071, *Bifidobacterium infantis* R0033, *Bifidobacterium breve* M-16V, and *Bifidobacterium infantis* M-63. The putative sortase-dependent proteins were identified by surveying the bifidobacterial sequences for a pentaglycine recognition motif (LPXTG in the case of sortase A- and sortase C- dependent proteins and [L/I/V][S/A]XTG in the case of sortase E-dependent proteins), followed by a hydrophobic membrane-spanning region, and positively charged residues at the C-terminus.

Supplementary Table 4: Putative sortase-dependent proteins in *Bifidobacterium breve* M-16V

ID	MW	Predicted function	GH family	Recognition motif (Sortase)	C-terminal positive charge
**BBRE1_0100**	64599	Cell surface protein	-	LPKTG (SrtA/ SrtC)	RARRKA
**BBRE1_0163**	25922	Hypothetical protein	-	VAFTG (SrtE)	YRRRQA
**BBRE1_0258**	69783	Hypothetical secreted protein	-	LPHTG (SrtA/ SrtC)	SRWEER
**BBRE1_0303**	131207	VWA domain-containing protein	-	LPVTG (SrtA/ SrtC)	WRKRQL
**BBRE1_0304**	53188	CNA-domain protein	-	LPLTG(SrtA/ SrtC)	KRALDA
**BBRE1_0618**	48470	Permease protein of ABC transporter system	-	LAATG (SrtE)	DSRKKRKGV
**BBRE1_1012**	21606	Hypothetical membrane spanning protein	-	LAVTG (SrtE)	HPRTTR
**BBRE1_1146**	194331	Cell surface protein	-	LASTG (SrtE)	AGK
**BBRE1_1442**	22998	Permease protein of ABC transporter system	-	IAKTG (SrtE)	RRA

MW= molecular weight of translated protein

Supplementary Table 5: Putative sortase-dependent proteins in *Bifidobacterium infantis* R0033

ID	MW	Predicted function	GH family	Recognition motif (Sortase)	C-terminal positive charge
**BINF_0103**	88585	Metallophosphoesterase	-	LSATG (SrtE)	KVVRRR
**BINF_0154**	9992	Hypothetical protein	-	VAATG (SrtE)	KVG
**BINF_0256**	67076	Histidine-type phosphatase	-	LARTG (SrtE)	VWRMRR
**BINF_0270**	70487	Cell-surface protein	-	LPVTG (SrtA/ SrtC)	VVRWRRRYDRFN
**BINF_0334**	66220	Hypothetical protein	-	LSNTG (SrtE)	AKH
**BINF_0423**	32833	Arabinogalactan endo-β-galactosidase	53	LSNTG (SrtE)	LRRKCS
**BINF_0478**	27122	Murein hydrolase transporter	-	LAVTG (SrtE)	RPRTTR
**BINF_0479**	21606	Hypothetical membrane spanning protein	-	IAPTG (SrtE)	LARRSI
**BINF_1579**	21080	Permease protein of ABC transporter system	-	LAATG (SrtE)	RSKARV
**BINF_1866**	124327	5'-nucleotidase	-	LPGTG (SrtA/ SrtC)	RRRQQR
***BINF_1976**	54241	Fimbrial subunit	-	LPLTG (SrtA/ SrtC)	FAKSRSTKRALNA
***BINF_1977**	85695	Fimbriae protein with VWA-domain	-	LPLTG (SrtA/ SrtC)	RKRQLV
**BINF_2214**	59826	Glycosyl hydrolase	unknown	LSATG (SrtE)	LSRKLS

MW= molecular weight of translated protein

*indicates protein associated with assemblage of fimbrial pili

Supplementary Table 6: Putative sortase-dependent proteins in *Bifidobacterium infantis* M-63

ID	MW	Predicted function	GH family	Recognition motif (Sortase)	C-terminal positive charge
**BINF2_0166**	83759	Penicillin-binding protein	-	ISSTG (SrtE)	NKR
**BINF2_0259**	66984	Hypothetical protein	-	LARTG (SrtE)	VWRMRR
**BINF2_0279**	70653	Cell surface protein	-	LPVTG (SrtA/ SrtC)	VVRWRRRYDRFN
**BINF2_0437**	32845	Arabinogalactan endo-β-galactosidase	53	LSNTG (SrtE)	LRRKCS
**BINF2_1235**	106050	Cell surface protein	-	LASTG (SrtE)	RNRRRA
**BINF2_1248**	14069	Cell surface protein	-	LPFTG (SrtA/ SrtC)	IRNRRRHGTRRR
**BINF2_1268**	100782	Hypothetical protein	-	LASTG (SrtE)	HVN
**BINF2_1270**	238421	Cell surface protein	-	LAKTG (SrtE)	RRRHAVTPRHGR
**BINF2_2163**	124368	5'-nucleotidase	-	LPGTG (SrtA/ SrtC)	RRRQQR
**BINF2_2469**	59826	Glycosyl hydrolase	unknown	LSATG (SrtE)	LSRKLS

MW= molecular weight of translated protein

Supplementary Table 7: Putative sortase-dependent proteins in *Bifidobacterium bifidum* R0071

ID	MW	Predicted function	GH family	Recognition motif (Sortase)	C-terminal positive charge
**BBIF1_0014**	13603	Cell wall anchoring protein	-	LSKTG (SrtE)	RKSGRF
**BBIF1_0020**	123042	α-L-arabinofuranosidase	62	LSHTG (SrtE)	FRRKRS
**BBIF1_0222**	23029	α-L-arabinofuranosidase	62	LSKTG (SrtE)	VARRRREPRR
**BBIF1_0227**	205512	1,2-α-L-fucosidase	95	VAKTG (SrtE)	RRKHSA
**BBIF1_0267**	17736	Hypothetical protein	-	VAKTG (SrtE)	VRKRATR
**BBIF1_0297**	206219	Endo-α-*N*-acetylgalactosaminidase	20	ISKTG (SrtE)	ARKRAE
**BBIF1_0307**	17635	α-L-arabinofuranosidase	62	LSATG (SrtE)	RRRRDI
***BBIF1_0313**	126084	Cell surface protein FimQ	-	LPMTG (SrtA/ SrtC)	RKRRLV
***BBIF1_0314**	55762	Fimbrial subunit FimP	-	LPKTG (SrtA/ SrtC)	YAKSRRTSRALR
**BBIF1_0347**	66830	Hypothetical protein	-	LSRTG (SrtE)	RPTSAR
**BBIF1_0450**	208523	Fibronectin type III	-	ISVTG (SrtE)	NRP
**BBIF1_0483**	188508	Hypothetical protein	-	LSKTG (SrtE)	VVRRRR
**BBIF1_0505**	206844	β-galactosidase	2	LSKTG (SrtE)	LRRKRS
**BBIF1_0589**	58936	Substrate-binding protein OppA	-	VAVTG (SrtE)	FRYASVTKS
**BBIF1_0822**	28898	Transposase subunit B	-	LPSTG (SrtA/ SrtC)	SRI
**BBIF1_0981**	113219	β-*N*-acetylhexosaminidase	20	VAETG (SrtE)	VRRQRR
**BBIF1_1173**	99824	Hypothetical protein	-	LSKTG (SrtE)	RRKHAI
**BBIF1_1299**	88827	Lipoprotein	-	LAHTG (SrtE)	GRRLRISRE

Supplementary Table 7: Putative sortase-dependent proteins in *Bifidobacterium bifidum* R0071

ID	MW	Predicted function	GH family	Recognition motif (Sortase)	C-terminal positive charge
**BBIF1_1306**	135311	Glycosyl hydrolase	unknown	IAATG (SrtE)	RKRRES
**BBIF1_1367**	158689	α-1-3,4-fucosidase	29	IAKTG (SrtE)	KRKSNR
**BBIF1_1382**	86609	Cation-transporting ATPase	-	VAMTG (SrtE)	QRAWLRSRR
**BBIF1_1436**	119514	Lacto-*N*-biosidase	20	LSATG (SrtE)	RRRSVR
**BBIF1_1494**	208333	β-*N*-hexosaminidase	20	LSKTG (SrtE)	ARSRRR
**BBIF1_1496**	167691	Calcineurin phosphoesterase	-	LSQTG (SrtE)	AVRTSKRADRIR
**BBIF1_1499**	72864	PTS system glucose-specific transporter	-	VAETG (SrtE)	IQR
**BBIF1_1506**	210337	Hyaluronidase	56	ISKTG (SrtE)	RRRREA
**BBIF1_1522**	176438	β-*N*-acetylhexosaminidase NagZ	20	VAKTG (SrtE)	RKRRI
**BBIF1_1584**	189580	Hypothetical protein	-	LSATG (SrtE)	AKR
**BBIF1_1620**	123721	NagC xylr-type transciptional regulator	-	LSNTG (SrtE)	LRKRIG
**BBIF1_1643**	80721	Hypothetical protein	-	LSHTG (SrtE)	RRRSRR
***BBIF1_1693**	57818	Fimbrial subunit FimA	-	LPLTG (SrtA/ SrtC)	KSRKASAIA
***BBIF1_1694**	38976	Cell surface protein FimB	-	LPLTG (SrtA/ SrtC)	AKRSRA
**BBIF1_1779**	189091	Exo-α-sialidase	33	VAKTG (SrtE)	RRRANR
**BBIF1_1780**	87015	Exo-α-sialidase	33	LSKTG (SrtE)	RRRSVH
***BBIF1_1806**	54908	Cna-protein B-type domain	-	LPGTG (SrtA/ SrtC)	LKRKNA
***BBIF1_1807**	264701	Hypothetical protein	-	LPDTG (SrtA/ SrtC)	RSGSSRIRKRGAHAS

MW= molecular weight of translated protein

*indicates protein associated with assemblage of fimbrial pili

Supplementary Figure 3

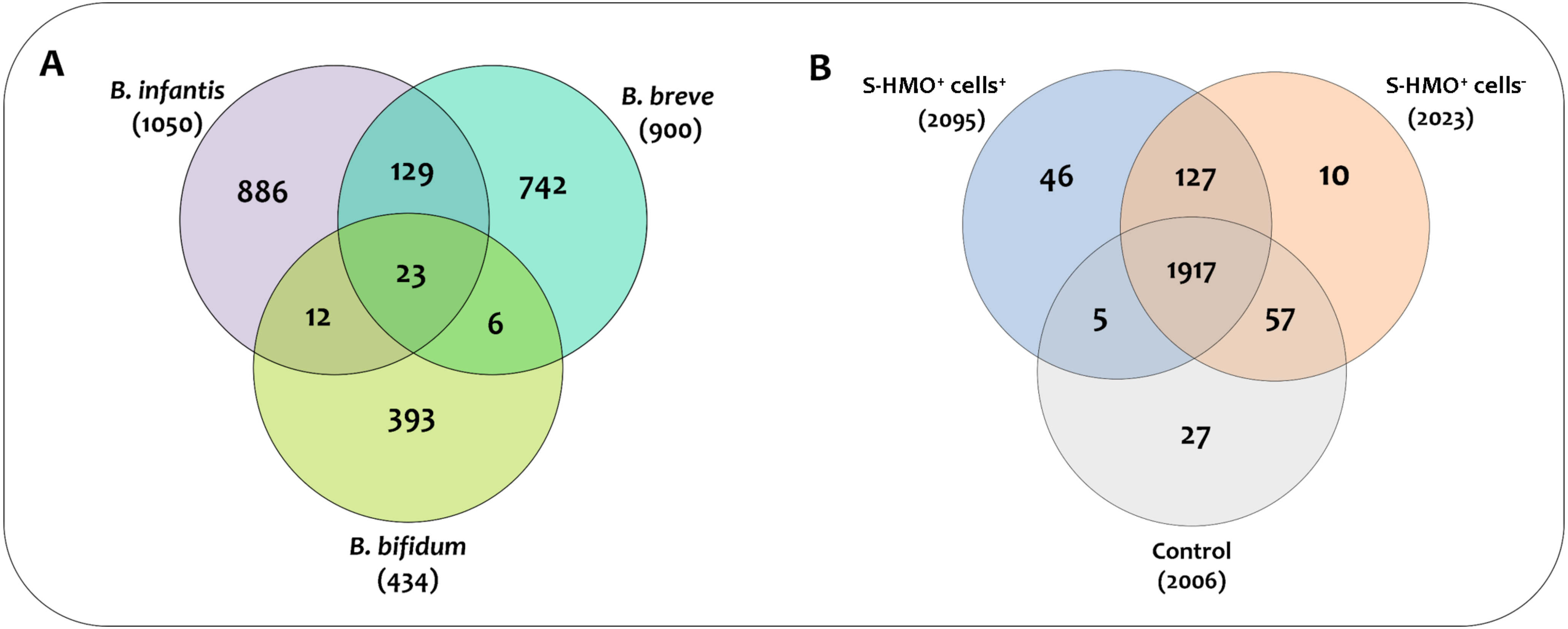

Figure S3: Numbers of bifidobacterial proteins detected by LC-MS/MS analysis in four-strain bacterial mixture following i) treatment with HMO and incubation with cells (S-HMO^{+} Cells^{+}), ii) incubation with cells without HMO-treatment (S-HMO^{-} Cells^{+}), iii) untreated bifidobacteria without incubation with HT-29 cells (control). Venn diagram shows distribution of protein database based on **A)** strain and **B)** sample group (total numbers of proteins detected in each sample group/ for each strain shown in brackets)

Supplementary Table 8: Proteins matched to *B. bifidum* found at significantly higher abundance/ uniquely detected after exposure to HT29-MTX intestinal cells alone or in combination with S-HMO

Locus	Protein function	fold change vs control	
		**S-HMO^{+}cells^{+}**	**S-HMO^{-}cells^{+}**
BBIF1_1522	β-*N*-acetylhexosaminidase NagZ (GH20)	**13.77**	**8.45**
BBIF1_0227	12-α-L-fucosidase (GH95)	**11.36**	**4.89**
BBIF1_0442	DNA-directed RNA polymerase subunit β RpoB	**10.99**	**5.23**
BBIF1_1523	50S ribosomal protein L17	**10.25**	**5.07**
BBIF1_1524	DNA-directed RNA polymerase subunit α	**9.99**	**5.35**
BBIF1_1806	Cna-protein B-type domain	**8.44**	**5.43**
BBIF1_1752	Polyphosphate kinase	**8.28**	**6.13**
BBIF1_1379	Nox NADH oxidase H2O-forming	**8.12**	**3.42**
BBIF1_1581	50S ribosomal protein L12	**8.03**	**4.41**
BBIF1_0505	β-galactosidase (GH2)	**7.89**	**4.03**
BBIF1_0274	PTS system *N*-acetylglucosamine-specific transporter subunit IIBC	**7.78**	**4.85**
BBIF1_1436	Lacto-*N*-biosidase (GH20)	**7.59**	**3.74**
BBIF1_1367	α-1-3,4-fucosidase (GH29)	**7.59**	**4.07**
BBIF1_0860	Glucose-1-phosphate adenylyltransferase glgc	**7.27**	**4.59**
BBIF1_0297	Endo-α-N-acetylgalactosaminidase	**7.20**	**2.72**
BBIF1_1534	50S ribosomal protein L18	**7.06**	**3.99**
BBIF1_1611	Ribonucleoside-diphosphate reductase subunit α nrde	**6.94**	**3.64**
BBIF1_1535	50S ribosomal protein L6	**6.90**	**3.93**
BBIF1_1309	Phosphoribosylformylglycinamidine synthase purl	**6.46**	**3.27**
BBIF1_1549	50S ribosomal protein L4	**6.42**	**3.68**
BBIF1_1544	30S ribosomal protein S3	**6.42**	**3.57**
BBIF1_0255	LSU ribosomal protein L11P	**6.23**	**3.63**
BBIF1_1547	50S ribosomal protein L2	**6.23**	**3.46**
BBIF1_0982	Threonyl-trna synthetase	**6.05**	**3.34**
BBIF1_1538	50S ribosomal protein L5	**5.91**	**3.39**
BBIF1_1127	Phenylalanyl-trna synthetase subunit β	**5.85**	**3.78**
BBIF1_1559	50S ribosomal protein L13	**5.81**	**3.39**

Supplementary Table 8: Proteins matched to *B. bifidum* found at significantly higher abundance/ uniquely detected after exposure to HT29-MTX intestinal cells alone or in combination with S-HMO

Locus	Protein function	fold change vs control	
		**S-HMO⁺cells⁺**	**S-HMO⁻cells⁺**
BBIF1_0356	30S ribosomal protein S16	**5.81**	**3.61**
BBIF1_0314	Fimbrial subunit FimP	**5.76**	**3.71**
BBIF1_1582	50S ribosomal protein L10	**5.72**	**3.24**
BBIF1_1013	UDP-glucose 4-epimerase gale	**5.70**	**3.70**
BBIF1_1301	Protein translation elongation factor G FusA	**5.62**	**3.25**
BBIF1_1198	Formate acetyltransferase	**5.52**	**3.19**
BBIF1_0682	Histidyl-trna synthetase	**5.42**	**3.65**
BBIF1_0615	Hypothetical protein	**5.28**	**3.43**
BBIF1_1545	50S ribosomal protein L22	**5.21**	**2.96**
BBIF1_1543	50S ribosomal protein L16	**5.19**	**3.05**
BBIF1_0199	Evolved β-galactosidase subunit α	**5.14**	**3.04**
BBIF1_1030	Protein translation elongation factor TS (Ef-Ts)	**5.12**	**3.30**
BBIF1_0313	Isopeptide protein S6	**5.12**	**2.79**
BBIF1_0838	SSU ribosomal protein S4P	**5.11**	**3.10**
BBIF1_0343	50S ribosomal protein L28	**5.10**	**2.37**
BBIF1_0560	L-lactate dehydrogenase	**5.06**	**2.87**
BBIF1_1506	Hyaluronidase	**5.04**	**2.53**
BBIF1_0582	Fructokinase	**5.00**	**3.29**
BBIF1_1531	50S ribosomal protein L15	**5.00**	**2.74**
BBIF1_1529	Adenylate kinase	**4.95**	**3.21**
BBIF1_1031	30S ribosomal protein S2P RpsB	**4.90**	**3.09**
BBIF1_1533	30S ribosomal protein S5	**4.90**	**3.05**
BBIF1_1021	Sugar ABC transporter substrate-binding protein	**4.89**	**2.99**
BBIF1_0483	Hypothetical protein	**4.87**	**2.90**
BBIF1_1300	Protein translation elongation factor Tu (Ef-Tu)	**4.84**	**3.18**
BBIF1_1715	Hypothetical protein	**4.83**	**3.13**
BBIF1_1001	Phosphoglycerate kinase	**4.80**	**2.85**

Supplementary Table 8: Proteins matched to *B. bifidum* found at significantly higher abundance/ uniquely detected after exposure to HT29-MTX intestinal cells alone or in combination with S-HMO

Locus	Protein function	fold change vs control	
		**S-HMO^{+}cells^{+}**	**S-HMO^{-}cells^{+}**
BBIF1_1610	Protein NrdI	**4.80**	1.57
BBIF1_0874	Branched-chain amino acid aminotransferase	**4.79**	**2.80**
BBIF1_0033	TrxB Thioredoxin reductase	**4.76**	**3.16**
BBIF1_1308	Phosphoribosylamidoimidazole-succinocarboxamide synthase	**4.73**	**3.09**
BBIF1_0966	GTP-binding protein	**4.71**	**3.11**
BBIF1_0804	Xylulose-5-phosphate Fructose-6-phosphate phosphoketolase	**4.70**	**2.89**
BBIF1_1194	Trigger factor PPiase	**4.70**	**2.84**
BBIF1_0853	CTP synthase PyrG	**4.61**	**3.20**
BBIF1_0002	Dnan DNA polymerase III β chain	**4.57**	**3.64**
BBIF1_1722	Phosphoenolpyruvate-protein phosphotransferase	**4.56**	**3.01**
BBIF1_0587	Glucosamine-6-phosphate isomerase NagB	**4.56**	**2.74**
BBIF1_0275	Homoserine dehydrogenase	**4.55**	**2.85**
BBIF1_1623	Type I multifunctional fatty acid synthase	**4.52**	**2.80**
BBIF1_0970	Phosphoribosylaminoimidazolecarboxamide formyltransferase	**4.49**	**2.88**
BBIF1_0027	Hypothetical protein	**4.48**	**2.91**
BBIF1_1634	ATP synthase subunit α	**4.48**	**2.89**
BBIF1_0589	Peptide ABC transporter substrate-binding protein OppA	**4.46**	0.88
BBIF1_0273	PTS system glucose-specific transporter subunit IIA	**4.45**	**2.82**
BBIF1_1652	Valyl-tRNA synthetase	**4.38**	**3.11**
BBIF1_1271	Protein translocase subunit SecA	**4.36**	**2.81**
BBIF1_0980	Hypothetical protein	**4.32**	**3.01**
BBIF1_1612	Ribonucleoside-diphosphate reductase β chain	**4.28**	1.74
BBIF1_1204	Peptidyl-prolyl cis-trans isomerase	**4.26**	**2.20**
BBIF1_0461	Membrane alanine aminopeptidase PepN	**4.21**	**2.66**
BBIF1_0855	ABC transporter ATP-binding protein	**4.20**	**2.49**
BBIF1_0991	Transketolase	**4.19**	**3.10**
BBIF1_1302	SSU ribosomal protein S7P	**4.18**	**2.50**

Supplementary Table 8: Proteins matched to *B. bifidum* found at significantly higher abundance/ uniquely detected after exposure to HT29-MTX intestinal cells alone or in combination with S-HMO

**Locus**	**Protein function**	**fold change vs control**	
		**S-HMO^{+}cells^{+}**	**S-HMO^{-}cells^{+}**
BBIF1_1294	Carbamoyl-phosphate synthase small chain	**4.17**	**2.36**
BBIF1_0776	Pyruvate kinase	**4.17**	**2.87**
BBIF1_0717	Glutamine synthetase GlnA	**4.14**	**2.50**
BBIF1_0681	Hypothetical protein	**4.06**	**2.67**
BBIF1_1673	Hypothetical protein	**4.04**	**3.07**
BBIF1_0017	NADP-specific glutamate dehydrogenase	**4.04**	**2.36**
BBIF1_0419	Phosphohydrolase	**4.03**	**3.31**
BBIF1_0032	Alkyl hydroperoxide reductase C22 protein	**4.00**	**2.51**
BBIF1_1809	ABC transporter ATP-binding protein	**4.00**	**2.53**
BBIF1_0778	Transcriptional regulator	**4.00**	**3.04**
BBIF1_1723	Phosphocarrier protein Hpr	**3.98**	**2.63**
BBIF1_0268	Lacto-*N*-biose phorylase	**3.95**	**2.68**
BBIF1_0379	Glucose-6-phosphate isomerase	**3.93**	**2.58**
BBIF1_1632	ATP synthase subunit β	**3.92**	**2.89**
BBIF1_0593	Xaa-pro aminopeptidase PepP	**3.91**	**3.06**
BBIF1_0961	Phosphoribosyl-ATP pyrophosphatase HisE	**3.89**	**2.71**
BBIF1_1296	Elongation factor P	**3.87**	**2.65**
BBIF1_0230	Formate--tetrahydrofolate ligase	**3.87**	**2.93**
BBIF1_0609	Glyceraldehyde 3-phosphate dehydrogenase Gap	**3.84**	**2.70**
BBIF1_0511	Hypothetical protein	**3.82**	**2.54**
BBIF1_0092	GlgP1 glycogen phosphorylase	**3.79**	**2.82**
BBIF1_1293	Carbamoyl-phosphate synthase large chain	**3.79**	1.86
BBIF1_1035	GMP reductase	**3.78**	**2.59**
BBIF1_1392	Prolyl-tRNA synthetase	**3.77**	**2.47**
BBIF1_1175	ABC transporter ATP-binding protein	**3.76**	**3.42**
BBIF1_1176	O-acetylhomoserine aminocarboxypropyltransferase	**3.75**	**3.20**
BBIF1_1470	Universal stress protein family	**3.74**	**3.07**

Supplementary Table 8: Proteins matched to *B. bifidum* found at significantly higher abundance/ uniquely detected after exposure to HT29-MTX intestinal cells alone or in combination with S-HMO

Locus	Protein function	fold change vs control	
		**S-HMO⁺cells⁺**	**S-HMO⁻cells⁺**
BBIF1_0890	Choloylglycine hydrolase	**3.64**	**2.70**
BBIF1_0647	Haloacid dehalogenase	**3.63**	**2.22**
BBIF1_1601	Aminopeptidase	**3.62**	**2.88**
BBIF1_0992	Transaldolase	**3.57**	**2.65**
BBIF1_1457	β -galactosidase (GH42)	**3.56**	1.11
BBIF1_1644	α-amylase	**3.55**	**2.74**
BBIF1_1478	Phosphoglycerate mutase	**3.54**	**2.42**
BBIF1_0726	Hypothetical protein	**3.52**	**2.30**
BBIF1_1077	Pyridoxamine 5-phosphate oxidase PdxH	**3.52**	**2.65**
BBIF1_1657	Transcription termination factor	**3.51**	**2.38**
BBIF1_0290	Aspartate aminotransferase	**3.50**	**2.86**
BBIF1_0632	Oligopeptide transport ATP-binding protein OppD	**3.50**	**2.20**
BBIF1_0633	Oligopeptide-binding protein OppA	**3.49**	**2.33**
BBIF1_1319	Phosphoribosylamine-glycine ligase	**3.48**	**2.36**
BBIF1_1398	Inosine-5-monophosphate dehydrogenase	**3.46**	**2.39**
BBIF1_1504	Phosphoglucomutase/phosphomannomutase	**3.45**	**2.87**
BBIF1_0655	DNA-binding protein HU	**3.45**	**2.28**
BBIF1_0240	Glutamyl-trna synthetase GltX	**3.39**	**2.43**
BBIF1_1625	Acetyl- propionyl-coa carboxylase subunit α AccC	**3.37**	**2.66**
BBIF1_0407	25-diketo-D-gluconic acid reductase	**3.37**	**2.18**
BBIF1_0416	Thioredoxin	**3.34**	**2.21**
BBIF1_1620	NagC xylr-type transciptional regulator	**3.33**	**2.71**
BBIF1_1645	Inorganic pyrophosphatase	**3.31**	**2.56**
BBIF1_1488	6-phosphogluconate dehydrogenase	**3.29**	**2.00**
BBIF1_0271	Arginyl-tRNA synthetase	**3.26**	**2.29**
BBIF1_1812	Protein-disulfide isomerase	**3.24**	**2.40**
BBIF1_1086	Ribose-phosphate pyrophosphokinase	**3.21**	**2.14**

Supplementary Table 8: Proteins matched to *B. bifidum* found at significantly higher abundance/ uniquely detected after exposure to HT29-MTX intestinal cells alone or in combination with S-HMO

Locus	Protein function	fold change vs control	
		**S-HMO^{+}cells^{+}**	**S-HMO^{-}cells^{+}**
BBIF1_1508	Ribose 5-phosphate isomerase	**3.15**	**2.17**
BBIF1_1047	Leucyl-tRNA synthetase LeuS	**3.14**	1.85
BBIF1_0463	Phosphoglucosamine mutase	**3.12**	**2.87**
BBIF1_0588	*N*-acetylglucosamine-6-phosphate deacetylase NagA	**3.10**	**2.32**
BBIF1_0740	Enolase	**3.08**	**2.15**
BBIF1_0669	60 kDa chaperonin GroeL	**3.06**	**3.10**
BBIF1_1389	Zinc metalloprotease PepO	**3.06**	**2.42**
BBIF1_0036	Ppc Phosphoenolpyruvate carboxylase	**3.01**	**2.46**
BBIF1_0424	3-isopropylmalate dehydrogenase	**3.00**	1.83
BBIF1_1161	GTP-dependent nucleic acid-binding protein EngD	**2.99**	**2.69**
BBIF1_1635	ATP synthase delta chain	**2.98**	**2.43**
BBIF1_0229	Dipeptidase A PepD	**2.96**	**2.28**
BBIF1_0803	GMP synthase (glutamine-hydrolyzing)	**2.96**	**2.24**
BBIF1_1617	Polynucleotide phosphorylase polyadenylase	**2.95**	**2.05**
BBIF1_0400	Pyridoxine biosynthesis protein	**2.86**	1.84
BBIF1_0886	Glycosyltransferase	**2.86**	**2.08**
BBIF1_0506	Blycyl-tRNA synthetase	**2.85**	1.55
BBIF1_1602	Hypothetical protein	**2.79**	**2.24**
BBIF1_0417	Hypothetical protein	**2.77**	**2.71**
BBIF1_0434	Hypothetical protein	**2.76**	1.83
BBIF1_1790	Chromate reductase NADPH-dependent FMN reductase	**2.74**	**2.08**
BBIF1_1663	Aspartyl glutamyl-trna amidotransferase subunit A	**2.74**	1.87
BBIF1_0386	ABC transporter permease	**2.73**	**2.90**
BBIF1_1324	ABC transporter substrate-binding protein	**2.70**	**2.52**
BBIF1_1495	Seryl-trna synthetase	**2.61**	1.86
BBIF1_1571	10 kda chaperonin GROES	**2.59**	**2.48**
BBIF1_0962	Ribulose-phosphate 3-epimerase	**2.57**	1.76

Supplementary Table 8: Proteins matched to *B. bifidum* found at significantly higher abundance/ uniquely detected after exposure to HT29-MTX intestinal cells alone or in combination with S-HMO

**Locus**	**Protein function**	**fold change vs control**	
		**S-HMO⁺cells⁺**	**S-HMO⁻cells⁺**
BBIF1_1156	Hypothetical protein	**2.54**	1.94
BBIF1_0375	Nicotinate phosphoribosyltransferase	**2.54**	**2.41**
BBIF1_0721	Peptidase family M20A protein	**2.51**	1.71
BBIF1_1019	Sugar ABC transporter permease	**2.49**	1.18
BBIF1_1451	Lactaldehyde reductase	**2.48**	**2.29**
BBIF1_1532	50S ribosomal protein L30	**2.48**	**2.21**
BBIF1_1784	Chaperone protein DnaK	**2.47**	**2.36**
BBIF1_0657	Adenylosuccinate lyase	**2.46**	1.86
BBIF1_0431	Galactose-1-phosphate uridylyltransferase	**2.45**	1.59
BBIF1_0788	Acetate kinase	**2.45**	1.69
BBIF1_1348	S-ribosylhomocysteinase	**2.44**	1.95
BBIF1_0946	UTP-glucose-1-phosphate uridylyltransferase	**2.42**	1.86
BBIF1_1018	Lacto-*N*-biose phorylase	**2.38**	1.45
BBIF1_1476	Phosphate ABC transporter	**2.28**	1.74
BBIF1_0672	Two-component response regulator	**2.18**	1.79
BBIF1_1555	Aldehyde-alcohol dehydrogenase 2	**2.16**	1.13
BBIF1_1681	Cell division protein FtsY	**2.12**	1.72
BBIF1_0237	ABC transporter substrate-binding protein	**2.07**	1.63
BBIF1_0235	Hypothetical protein	**2.04**	1.28
BBIF1_0475	Exodeoxyribonuclease VII small subunit	**2.02**	1.19
BBIF1_0059	Dtdp-rhamnosyl transferase RfbF	**2.01**	1.49
BBIF1_1303	SSU ribosomal protein S12P	1.70	1.56
BBIF1_0020	α-L-arabinofuranosidase (GH43)	Uniquely detected	
BBIF1_0183	Hypothetical protein	Uniquely detected	
BBIF1_0196	Ketol-acid reductoisomerase 2-dehydropantoate 2-reductase	Uniquely detected	
BBIF1_0262	LacI family transcriptional regulator	Uniquely detected	
BBIF1_0284	Ribonuclease G	Uniquely detected	

Supplementary Table 8: Proteins matched to *B. bifidum* found at significantly higher abundance/ uniquely detected after exposure to HT29-MTX intestinal cells alone or in combination with S-HMO

**Locus**	**Protein function**	**fold change vs control**	
		**S-HMO$^+$cells$^+$**	**S-HMO$^-$cells$^+$**
BBIF1_0360	Signal recognition particle subunit FFH SRP54	Uniquely detected	
BBIF1_0368	Hypothetical protein	Uniquely detected	
BBIF1_0377	Xanthosine triphosphate pyrophosphatase	Uniquely detected	
BBIF1_0399	Long-chain-fatty-acid-CoA ligase	Uniquely detected	
BBIF1_0477	Anaerobic ribonucleoside-triphosphate reductase	Uniquely detected	
BBIF1_0565	D-3-phosphoglycerate dehydrogenase	Uniquely detected	
BBIF1_0585	Copper homeostasis protein	Uniquely detected	
BBIF1_0634	Aminopeptidase	Uniquely detected	
BBIF1_0643	Dihydroxy-acid dehydratase	Uniquely detected	
BBIF1_0645	S-adenosylmethionine synthetase	Uniquely detected	
BBIF1_0718	Hypothetical protein	Uniquely detected	
BBIF1_0731	2-C-methyl-D-erythritol 4-phosphate cytidylyltransferase	Uniquely detected	
BBIF1_0843	Alanyl-tRNA synthetase	Uniquely detected	
BBIF1_0916	Aspartate carbamoyltransferase	Uniquely detected	
BBIF1_0918	Dihydroorotase PyrC	Uniquely detected	
BBIF1_0972	Succinyl-CoA synthetase subunit α	Uniquely detected	
BBIF1_0975	YajC Protein translocase subunit	Uniquely detected	
BBIF1_1015	*N*-acetylhexosamine-1-kinase NahK	Uniquely detected	
BBIF1_1128	Phenylalanyl-tRNA synthetase α chain	Uniquely detected	
BBIF1_1135	Uracil PyrP	Uniquely detected	
BBIF1_1272	Ribosome-associated factor Y	Uniquely detected	
BBIF1_1362	B-galactosidase	Uniquely detected	
BBIF1_1467	Dihydrofolate reductase	Uniquely detected	
BBIF1_1490	Oxppcycle protein OpcA	Uniquely detected	
BBIF1_0020	A-L-arabinofuranosidase (GH43)	Uniquely detected	
BBIF1_0115	Peptide methionine sulfoxide reductase MsrA MsrB	Uniquely detected	

Supplementary Table 8: Proteins matched to *B. bifidum* found at significantly higher abundance/ uniquely detected after exposure to HT29-MTX intestinal cells alone or in combination with S-HMO

**Locus**	**Protein function**	**fold change vs control**	
		**S-HMO$^+$cells$^+$**	**S-HMO$^-$cells$^+$**
BBIF1_0183	Hypothetical protein	Uniquely detected	
BBIF1_0196	Ketol-acid reductoisomerase 2-dehydropantoate 2-reductase	Uniquely detected	
BBIF1_0262	LacI family transcriptional regulator	Uniquely detected	
BBIF1_0284	Ribonuclease G	Uniquely detected	
BBIF1_0360	Signal recognition particle subunit FFH SRP54	Uniquely detected	
BBIF1_0368	Hypothetical protein	Uniquely detected	
BBIF1_0377	Xanthosine triphosphate pyrophosphatase	Uniquely detected	
BBIF1_0399	Long-chain-fatty-acid-CoA ligase	Uniquely detected	
BBIF1_0477	Anaerobic ribonucleoside-triphosphate reductase	Uniquely detected	
BBIF1_0565	D-3-phosphoglycerate dehydrogenase	Uniquely detected	
BBIF1_0585	Copper homeostasis protein	Uniquely detected	
BBIF1_0634	Aminopeptidase	Uniquely detected	
BBIF1_0643	Dihydroxy-acid dehydratase	Uniquely detected	
BBIF1_0645	S-adenosylmethionine synthetase	Uniquely detected	
BBIF1_0718	Hypothetical protein	Uniquely detected	
BBIF1_0731	2-C-methyl-D-erythritol 4-phosphate cytidylyltransferase	Uniquely detected	
BBIF1_0843	Alanyl-tRNA synthetase	Uniquely detected	
BBIF1_0916	Aspartate carbamoyltransferase	Uniquely detected	
BBIF1_0918	Dihydroorotase PyrC	Uniquely detected	
BBIF1_0972	Succinyl-coa synthetase subunit α	Uniquely detected	
BBIF1_0975	YajC Protein translocase subunit	Uniquely detected	
BBIF1_1015	*N*-acetylhexosamine-1-kinase NahK	Uniquely detected	
BBIF1_1028	Uridylate kinase	Uniquely detected	
BBIF1_1128	Phenylalanyl-trna synthetase α chain	Uniquely detected	
BBIF1_1135	Uracil ByrP	Uniquely detected	
BBIF1_1272	Ribosome-associated factor Y	Uniquely detected	

Supplementary Table 8: Proteins matched to *B. bifidum* found at significantly higher abundance/ uniquely detected after exposure to HT29-MTX intestinal cells alone or in combination with S-HMO

Locus	Protein function	fold change vs control	
		S-HMO^{+}cells^{+}	S-HMO^{-}cells^{+}
BBIF1_1362	β-galactosidase	Uniquely detected	
BBIF1_1467	Dihydrofolate reductase	Uniquely detected	
BBIF1_1490	Oxppcycle protein OpcA	Uniquely detected	
BBIF1_0257	Peptidyl-prolyl cis-trans isomerase	Uniquely	Not detected
BBIF1_0260	Thioredoxin protein	Uniquely	Not detected
BBIF1_0299	Hypothetical protein	Uniquely	Not detected
BBIF1_0485	ATP-binding protein of ABC transporter system	Uniquely	Not detected
BBIF1_0577	UDP-*N*-acetylmuramate--alanine ligase MurC	Uniquely	Not detected
BBIF1_0752	Sensory transduction protein kinase	Uniquely	Not detected
BBIF1_0917	Sspartate carbamoyltransferase regulatory subunit	Uniquely	Not detected
BBIF1_0942	Haloacid dehalogenase	Uniquely	Not detected
BBIF1_0947	Hypothetical protein	Uniquely	Not detected
BBIF1_0972	Succinyl-CoA synthetase subunit alpha	Uniquely	Not detected
BBIF1_1002	Hypothetical protein	Uniquely	Not detected
BBIF1_1033	Long-chain-fatty-acid-CoA ligase	Uniquely	Not detected
BBIF1_1038	Long-chain-fatty-acid-CoA ligase	Uniquely	Not detected
BBIF1_1268	1-Acyl-sn-glycerol-3-phosphate acyltransferase	Uniquely	Not detected
BBIF1_1270	Anthranilate phosphoribosyltransferase	Uniquely	Not detected
BBIF1_1275	Recombinase A	Uniquely	Not detected
BBIF1_1307	Phosphoribosylglycinamide formyltransferase PurT	Uniquely	Not detected
BBIF1_1318	Phosphoribosylformylglycinamidine cyclo-ligase PurM	Uniquely	Not detected
BBIF1_1336	Phosphoribosylaminoimidazole carboxylase catalytic subunit	Uniquely	Not detected
BBIF1_1482	Tetratricopeptide tPR_2 repeat protein	Uniquely	Not detected
BBIF1_1756	Glycosyltransferase	Uniquely	Not detected
BBIF1_0115	Peptide methionine sulfoxide reductase MsrA MsrB	Not detected	Uniquely
BBIF1_0418	Hydrolase	Not detected	Uniquely

Supplementary Table 8: Proteins matched to *B. bifidum* found at significantly higher abundance/ uniquely detected after exposure to HT29-MTX intestinal cells alone or in combination with S-HMO

**Locus**	**Protein function**	**fold change vs control**	
		**S-HMO^{+}cells^{+}**	**S-HMO^{-}cells^{+}**
BBIF1_0524	Hypothetical protein	Not detected	Uniquely
BBIF1_0705	ATP-binding Mrp protein	Not detected	Uniquely
BBIF1_1028	Uridylate kinase	Not detected	Uniquely
BBIF1_1505	Pyridine nucleotide-disulfide oxidoreductase family protein	Not detected	Uniquely
BBIF1_1510	Hypothetical protein	Not detected	Uniquely
BBIF1_1539	50S ribosomal protein L24	Not detected	Uniquely

Protein abundance evaluated using label-free quantitative proteomic analysis

Significant changes in protein abundance determined using Persus (v 1.6.2.2)

Values marked in **bold** indicate proteins with fold change ≥2

Supplementary Table 9: Proteins matched to *B. infantis* found at significantly higher abundance/ uniquely detected after exposure to HT29-MTX intestinal cells alone or in combination with S-HMO

**Locus**	**Protein function**	**fold change vs control**	
		S-HMO^{+}cells^{+}	S-HMO^{-}cells^{+}
BINF2_0342	Hypothetical protein	**2.48**	1.05
BINF2_0386	Riboflavin synthase β subunit	1.97	1.65
BINF2_0574	Leucine- isoleucine- valine- threonine- and alanine-binding	1.69	0.60
BINF2_0577	Branched-chain amino acid transport ATP-binding protein livG	1.79	0.69
BINF2_0876	Sugar kinase	**25.54**	**2.74**
BINF2_0878	Glucosamine-6-phosphate isomerase	**2.73**	0.72
BINF2_0879	*N*-acetylglucosamine-6-phosphate deacetylase	**2.81**	0.70
BINF2_1926	SSU ribosomal protein S12P	1.70	1.56
BINF2_2055	Hypothetical protein	**2.76**	1.83
BINF2_2172	Phosphotransferase	1.93	0.94
BINF2_2174	Sugar ABC transporter permease	**2.49**	1.18
BINF2_2225	30S ribosomal protein S17	**8.40**	**6.37**
BINF2_2242	50S ribosomal protein L13	**2.47**	1.29
BINF2_2305	Fucose transport protein	**2.62**	0.48
BINF2_2308	Short chain dehydrogenase	**10.95**	0.30
BINF2_2309	Mandelate racemase	**21.49**	1.49
BINF2_2333	Galactoside transport protein	1.92	0.60
BINF2_2345	ABC transporter substrate binding component	**4.68**	0.72
BINF2_2346	ABC transporter permease	**3.54**	0.78
BINF2_2347	ABC transporter permease	**5.91**	0.92
BINF2_2348	ABC transporter substrate binding component	**4.06**	0.68
BINF2_2349	Sialidase	**2.21**	0.67
BINF2_2350	Dihydrodipicolinate synthase	**2.16**	0.70
BINF2_2351	ABC transporter substrate binding component	1.89	0.64
BINF2_2422	Aminopeptidase C	**2.11**	0.84
BINF2_0170	DNA polymerase III delta subunit	Uniquely detected	
BINF2_1693	Uracil-xanthine transport protein	Uniquely detected	

Protein abundance evaluated using label-free quantitative proteomic analysis,

Significant changes in protein abundance determined using Persus (v 1.6.2.2). Values marked in **bold** indicate proteins with fold change ≥2

Supplementary Table 10: Proteins matched to *B. breve* found at significantly higher abundance/ uniquely detected after exposure to HT29-MTX intestinal cells alone or in combination with S-HMO

Locus	Protein encoded	fold change vs control	
		S-HMO$^+$cells$^+$	S-HMO$^-$cells$^+$
BBREV_01643	30S ribosomal protein S17	**8.40**	**6.37**
BBREV_01523	α-N-acetylglucosaminephosphotransferase	**3.23**	**2.49**
BBREV_00420	Hypothetical protein	**2.76**	1.83
BBREV_00353	LSU ribosomal protein L19P	**2.49**	1.80
BBREV_01561	β-galactosidase	**2.29**	0.58
BBREV_00100	Cell surface protein	**2.11**	1.07
BBREV_00616	Hypothetical membrane spanning protein	1.92	1.26
BBREV_01250	*N*-acetylglucosamine-6-phosphate deacetylase	1.70	0.75
BBREV_01525	Branched-chain amino acid transport ATP-binding protein	1.65	0.61
BBREV_01251	Glucosamine-6-phosphate isomerase	1.48	0.62
BBREV_01529	Leucine- isoleucine- valine- threonine- and alanine-binding	1.43	0.65
BBREV_00988	Prolipoprotein diacylglyceryl transferase	Uniquely detected	
BBREV_00092	Transport protein	Uniquely detected	

Protein abundance evaluated using label-free quantitative proteomic analysis

Significant changes in protein abundance determined using Persus (v 1.6.2.2)

Values marked in **bold** indicate proteins with fold change ≥2
